# Pentacyclic Triterpenoids Inhibit IKKβ Mediated Activation of NF-κB Pathway: *In Silico* and *In Vitro* Evidences

**DOI:** 10.1371/journal.pone.0125709

**Published:** 2015-05-04

**Authors:** Kalpesh R. Patil, Purusottam Mohapatra, Harun M. Patel, Sameer N. Goyal, Shreesh Ojha, Chanakya N. Kundu, Chandragouda R. Patil

**Affiliations:** 1 Department of Pharmacology, H. R. Patel Institute of Pharmaceutical Education and Research, Shirpur, Dist- Dhule, Maharashtra, India; 2 Cancer Biology Laboratory, KIIT School of Biotechnology, KIIT University, Bhubaneswar, Odisha, India; 3 Department of Pharmaceutical Chemistry, R. C. Patel Institute of Pharmaceutical Education and Research, Shirpur, Dist- Dhule, Maharashtra, India; 4 Department of Pharmacology, R. C. Patel Institute of Pharmaceutical Education and Research, Shirpur, Dist- Dhule, Maharashtra, India; 5 Department of Pharmacology and Therapeutics, College of Medicine and Health Sciences, United Arab Emirates University, Al-Ain, United Arab Emirates, UAE; Henry Ford Health System, UNITED STATES

## Abstract

Pentacyclic Triterpenoids (PTs) and their analogues as well as derivatives are emerging as important drug leads for various diseases. They act through a variety of mechanisms and a majority of them inhibit the nuclear factor kappa-beta (NF-κB) signaling pathway. In this study, we examined the effects of the naturally occurring PTs on IκB kinase-β (IKKβ), which has great scientific relevance in the NF-κB signaling pathway. On virtual screening, 109 PTs were screened through the PASS *(prediction of activity spectra of substances)* software for prediction of NF-κB inhibitory activity followed by docking on the NEMO/IKKβ association complex (PDB: 3BRV) and testing for compliance with the softened Lipinski’s Rule of Five using Schrodinger (LLC, New York, USA). Out of the projected 45 druggable PTs, Corosolic Acid (CA), Asiatic Acid (AA) and Ursolic Acid (UA) were assayed for IKKβ kinase activity in the cell free medium. The UA exhibited a potent IKKβ inhibitory effect on the hotspot kinase assay with IC50 of 69 μM. Whereas, CA at 50 μM concentration markedly reduced the NF-κB luciferase activity and phospho-IKKβ protein expressions. The PTs tested, attenuated the expression of the NF-κB cascade proteins in the LPS-stimulated RAW 264.7 cells, prevented the phosphorylation of the IKKα/β and blocked the activation of the Interferon-gamma (IFN-γ). The results suggest that the IKKβ inhibition is the major mechanism of the PTs-induced NF-κB inhibition. PASS predictions along with *in-silico* docking against the NEMO/IKKβ can be successfully applied in the selection of the prospective NF-κB inhibitory downregulators of IKKβ phosphorylation.

## Introduction

Nuclear factor kappa B (NF-κB) is a family of ubiquitous transcription factors comprising five related elements, namely p50, p52, RelA, RelB and c-Rel [1–3]. NF-κB plays an important role in mediating the immune responses by regulating the expression of various proinflammatory and immune-regulatory cytokines, inducible nitric oxide synthase, cyclo-oxgenase-2, growth factors and the apoptotic cascade. The pathological derangement in the NF-κB signaling is linked with the onset and progression of inflammation and associated autoimmune diseases as well as cancer [4,5].

In NF-κB signal activation two pathways namely classical (canonical pathway) and an alternative pathway (non-canonical pathway) are involved [6–9]. The, NF-κB complex is present in the cytoplasm in an inactive state with the inhibitory κB proteins (IκB). The inhibitory κB protein kinases (IKKs) are essential components of the signaling pathways by which NF-κB is activated in response to the pro-inflammatory stimuli. The upstream stimuli, like lipopolysaccharide (LPS), tumor necrosis factor alpha (TNF-α) and Interleukin-1 (IL-1) activate the IκB kinase (IKK) complex, consisting of catalytic IKKα and IKKβ subunits along with the regulatory subunit IKKγ termed NEMO (NF-κB Essential Modulator) [10,11]. In both pathways; classical and alternative IKK activation is a common regulatory step initiating the NF-κB signaling. Although, both the catalytic units of the IKK complex have the capacity to phosphorylate IκB, the IKKβ plays a dominant role in activating the NF-κB signaling in response to inflammatory stimuli [12,13]. The IKK mediated phosphorylation and proteasomal degradation of the IκB inhibitor trigger the activation and subsequent translocation of the NF-κB to the cellular nucleus. The translocated NF-κB elicits the expression of the target genes that encode several pro-inflammatory cytokines participating in the acute inflammatory response [14]. The transcriptional activity of NF-κB induces the expression of IκBα gene and generates IκBα, which consequently sequesters the NF-κB subunits and terminates the transcriptional activity of NF-κB [15].

The IKKβ plays a central role in the inflammatory stimuli through the regulation of the NF-κB signaling. Therefore, it is an attractive target for the therapeutic intervention in the various immune-inflammatory pathological conditions, such as inflammatory bowel disease (IBD), rheumatoid arthritis and muscular dystrophy [16–18]. Several IKKβ inhibitors are being investigated for their druggability [19,20]. However, the unavailability of the crystal structure of the IKKβ had halted the discovery of new inhibitors through the virtual screening of the compound libraries. Until the recent past, the IKKβ inhibitors had been identified through the pharmacophore-based or high-throughput screening approaches [12,21,22]. In 2011, the X-ray co-crystal structure of the IKKβ with the reference inhibitor ((4-{[4-4-chlorophenyl)pyrimidin-2-yl]amino}phenyl[4-(2-hydroxyethyl)piperazin-1-yl] methanone (PDB: 3RZF) was reported as an updated structure of the IKKβ [12,23]. Before this report, the structure of a NEMO-IKKβ association complex (PDB: 3BRV) was used for docking studies of a steroidal phytoconstituent, Withaferin A [10]. The screening of a library of 90000 compounds from the ZINC natural products database against the updated structure of the IKKβ yielded a benzoic acid derivative as the most potent IKKβ inhibitor having an inhibitory concentration (IC_50_) ~ 50 μM [12]. Likewise, Huang et al. [24] combined structure-based and ligand-based methods using the co-crystal structure of IKKβ and identified AI-898/12177 and NSC 302961 as the potent inhibitors from among the 162 known IKK inhibitors. However, more attractive chemical scaffolds and pharmacophores from natural resources remain to be screened as the NF-κB modulators to yield the leads for the discovery of novel IKKβ inhibitors.

The NF-κB is an important transcription factor involved critically in the pathophysiology of many diseases including cancer and immune-inflammatory disorders. Inhibition of NF-κB activation is a promising therapeutic approach to fight against several human diseases. The aspirin and glucocorticoids are regarded as the inhibitors of NF-κB activity [25,26]. The inhibitors of NF-κB pathway can reduce the inflammatory response and potentiates the effects of cancer chemotherapy [27]. The extensive research on NF-κB inhibitors is crucially needed for the development of highly efficacious, safer and economical anti-inflammatory and anticancer drugs [28]. The pentacyclic triterpenoids (PTs) are multifunctional molecules having the ability to inhibit NF-κB signaling. The PTs have attracted attention due to their ability to interact with multiple biological targets. These secondary plant metabolites and their semisynthetic derivatives exert dose dependent pharmacological actions and few of them have entered the later stages of clinical trials [29]. Many PTs including oleanolic acid, ursolic acid, corosolic acid, asiatic acid and glycyrrhizic acid have been proved to possess therapeutically useful biological profiles. PTs are components of routine foodstuffs and are relatively nontoxic. These properties of PTs provide the fertile ground for the development of PTs based phytopharmaceuticals [30–32]. PTs are rich source of lead compounds for drug development and worth for further systematic evaluations through preclinical and clinical trials [33]. Several anti-inflammatory triterpenoids derived from natural sources inhibit the NF-κB signaling [25,34–38]. Certain studies attributed the anti-inflammatory activities of the PTs at least in part, to IKKβ inhibition [38,39]. Such an inhibition of the IKKβ and the subsequent blockade of the NF-κB signaling by the PTs could lead to the emergence of therapeutic agents for inflammatory diseases [32,37,40–44].

The virtual screening of synthetic compound libraries is a routine that accelerates and economizes the drug discovery process through the rapid identification of hits and a reduction in the biological screening of irrelevant compounds. However, virtual screening of the phytochemicals is believed to be a major source of drug discovery and in particular anticancer agents are still underscored and in this case its role rather remains underestimated [45]. The PASS (prediction of activity spectra of substances) is an important *in-silico* tool used for predicting the biological activity spectra of natural and synthetic substances. A recent review by Filimonov et al. [46] justifies the efficacy of PASS software in predicting biological activity spectrum of various substances. The PASS has been used to screen the compound libraries for exploration of their hidden biological potentials and to determine the priorities for *in vitro* and *in vivo* biological testing [45–47]. The utility of chemo-bioinformatics resources and *in-silico* molecular docking using GLIDE software from Schrodinger are widely acclaimed as validated tools for *in-silico* screening of natural products [47,48].

The present study was designed to determine whether the naturally occurring prospective NF-κB inhibitory PTs exert IKKβ inhibitory activity as well as to identify the potent IKKβ inhibitors. Here, we investigated the inhibitory effects of the PTs on the IKKβ and ultimately the NF-κB activity. An assembled dataset of the PTs was screened through the PASS software to predict the NF-κB inhibitory activity. Moreover, molecular docking analysis was performed to elucidate the binding modes of the PTs with the NEMO/IKKβ complex. Three potential compounds, Corosolic Acid (CA), Asiatic Acid (AA) and Ursolic Acid (UA) included under the list of ranked active compounds based on their predicted activity were extensively studied through *in vitro* assays. The compounds were evaluated by standard techniques such as hotspot IKKβ kinase assay, protein expression by western blot, NF-κB luciferase reporter assay and interferon gamma (IFN-γ) expression in LPS stimulated RAW 264.7 macrophages. We have validated whether the sequential application of PASS followed by the molecular docking of the PASS-predicted compounds yielded the most effective IKKβ inhibitory triterpenoids. The accuracy of the prediction was reconfirmed through *in vitro* assays.

## Materials and Methods

### Virtual screening protocol

A dataset consisting of 109 naturally occurring pentacyclic triterpenoids believed to be prospective NF-κB inhibitor (S1 Table) were processed through the PASS software. Further, the compounds predicted as NF-κB inhibitors by PASS software with a probability of activity, Pa > 0.3 were chosen and set in the descending order of predicted probability. These compounds were docked on IKKβ crystal structure retrieved from the Protein Data Bank [PDB: 3BRV] using the molecular docking software Glide (Version 5.5, Schrodinger, LLC, New York, USA, 2009) to determine its binding potential. The compounds having the highest docking scores were tested using the QikProp 3.2 utility for compliance by the softened Lipinski’s Rule of Five to evaluate drug likeness [49]. The obtained *in silico* results were further confirmed by the *in vitro* assays and the IC_50_ was determined for the compounds included within the predicted IKKβ inhibitory activity spectrum, using the hotspot kinase assay. The cytotoxic concentration was determined by the 3-(4,5-dimethylthiazol-2-yl)-2,5-diphenyl tetrazolium bromide (MTT) assay using the RAW 264.7 cells. Further, these compounds were tested for their effects on the NF-κB, phosphorylated IKKβ, IKKα, Akt and C-Jun protein expressions using western blot. The NF-κB and IFN-γ inhibitory activity was measured by using luciferase reporter assay and indirect ELISA, respectively.

### Pentacyclic triterpenoids library (Dataset)

The objective of search strategy was to prepare a compound library of the PTs through retrieval of published literature reporting the molecular mechanisms involved in anti-inflammatory, anti-arthritic and anticancer activities. We used search engines including MEDLINE, EMBASE and Cochrane library prior to December 2013 to identify information regarding the role of the PTs in suppressing NF-κB signaling. The search strategy involved the use of appropriate MeSH terms like PTs and/or NF-κB and/or IκB Kinase. The structures of the compounds were either downloaded from the NCBI (http://pubchem.ncbi.nlm.nih.gov/search/search.cgi) or drawn using Chem Biodraw 11.0. The structures were verified against the published literature [29,34,38]. The compounds retrieved through systematic search were converted to ‘.mol’ format using the Chem Biodraw 11.0 software. The three-dimensional (3D) conversion and minimization were performed using LigPrep 2.3 (Merck Molecular Forces Field; MMFF) [50]. The conformers were generated using a rapid torsion angle search approach followed by the minimization of each structure generated using the MMFFs, with an implicit GB/SA solvent model. A maximum of 1000 conformers were generated per structure using a pre-process minimization of 1000 steps and post process minimization of 500 steps. Each minimized conformer was filtered through a relative energy window of 50 kJ mol−1 and a minimum atom deviation of 1.00 Å, to set an energy threshold relative to the lowest energy conformer. The conformers that were higher in energy than the given threshold were discarded. The distance between all the pairs of the corresponding heavy atoms needed to be below 1.00 Å to consider the two conformers identical. This threshold was applied only after the energy difference threshold and if the two conformers were within 1 kcal mol−1 of each other.

### Prediction of activity spectrum using PASS

The *in silico* prediction of the NF-κB inhibitory activity was conducted using the PASS (http://www.pharmaexpert.ru/PASSOnline) software version: 2.0. The PASS estimations of biological activity spectra of new compounds are based on the structure activity relationships knowledgebase (SAR Base) which accumulate the results of the training set analysis [51]. The PASS training set includes known biologically active substances (drugs, drug candidates, pharmaceutical leads and toxic compounds). New information regarding biologically active compounds is discovered regularly and the special informational search and information analyzed is further used for updating and correcting the PASS training set. Leaving one out cross-validation (LOO CV) for the whole PASS training set, which includes 205873 substances, provides 95% of the PASS prediction accuracy during the trial periods. This software estimates the predicted activity spectrum of a compound in terms of probable activity (Pa) and probable inactivity (Pi).

### Docking of the selected PTs on IKKβ

Structure based virtual screening was performed on the Molecular Modelling Interface (Schrodinger, LLC, New York, USA) installed on Core 2 Duo Quad PC with Windows XP operating system using Glide 5.5 and QikProp 3.2 [52,53]. The crystal structure of the NEMO/IKKβ association domain was obtained from the RCSB Protein Data Bank (PDB) with the accession code 3BRV (http://www.rcsb.org/pdb/explore/explore.do?structureId=3BRV). The protein structure consists of a 4-helix bundle of the NEMO and IKKβ domains, each containing two chains B, D and A, C, respectively. To study the interaction of the compounds for the IKKβ subunit, the molecules were docked on the chains representing the NEMO/IKKβ subunit by using Maestro 9.0 (Schrodinger, LLC, New York, USA). The protein preparation was performed using the ‘protein preparation wizard’ in Maestro 9.0 in two steps, viz. preparation and refinement. After ensuring the chemical correctness, the water molecules in the crystal structures were deleted and hydrogen was added, wherever necessary. Using the optimized potential for liquid simulation (OPLS) the force field energy of the crystal structure was minimized [54]. Ligands were built using Maestro build panel and were prepared by the LigPrep 2.3 module, which produced a low-energy conformer of ligands following the OPLS force field. The low energy conformation of ligands was selected and docked into the grid generated from the protein structures using the extra precision (XP) docking mode. The final evaluation was done using the glide score (docking score) and the number of hydrogen bond interactions. The single best pose was generated as the output for a particular ligand.

### Determination of compliance with the ‘Softened Lipinski’s Rule of Five’

All the PTs having highest docking scores were tested for compliance with the softened Lipinski’s Rule of Five to evaluate drug likeness and established the *in silico* ADME parameters using QikProp 3.2 [49,53]. We analyzed the physically significant descriptors and pharmaceutically relevant properties of PTs, among which were molecular weight, LogP, H-bond donors and H-bond acceptors, according to Lipinski’s Rule of Five. Generally, Lipinski’s parameters determine either poor oral absorption or membrane permeability that occurs when the molecules evaluated present values higher than five H-bond donors (HBD), 10 H-bond acceptors (HBA), molecular weight (MW) > 500 Da and LogP (cLogP) > 5 (as per the Lipinski’s Rule of Five) [55]. The total number of violations of Lipinski’s Rule of Five lies between 0 and 4. The molecules having violation scores greater than one were considered to be marginal for further development [49,56,57]. Recently, Petit et al. [49] emphasized to soften the ‘Rule of Five’ procedures, which are based on replacing the sharp utility function of the classical Lipinski’s Rule of Five. Considering the importance of the ‘Rule of Five’ in predicting drug likeliness and avoiding the over filtering of the compounds, we applied the softened Rule of Five. The compounds which violated more than one of the ‘Lipinski’s Rule of Five’ were excluded from the further study.

### High throughput kinase (IKKβ) assay

The *in vitro* activity profiling of CA, AA and UA was performed using the Hot Spot assay platform, as described earlier [58,59]. Briefly, the IKKβ and IKKtide [KKKKERLLDDRHDSGLDSMKDEE] peptide, representing the kinase and substrate pair were prepared along with the required cofactors in the reaction buffer (20 mM HEPES, pH 7.5, 10 mM MgCl_2_, 1 mM EGTA, 0.02% BRIJ 35, 0.02 mg/ml BSA, 0.1 mM Na_3_VO_4_, 2 mM DTT, 1% DMSO). The test compounds CA, AA and UA were studied at 10 dose levels to determine the IC_50_ value with three fold serial dilution starting at 100 μM and the control compound staurosporine was studied at the three fold serial dilution starting at 20 μM. The compounds were dissolved in DMSO and added into the reaction mixture. After 20 min, a mixture of ATP (Sigma, USA) and 33P ATP (Perkin Elmer, Massachusetts, USA) was added to a final concentration of 10 μM, to initiate the reaction process. The reaction was performed at 25°C for 120 min followed by spotting of the reaction onto a P81 ion exchange filter paper (Whatman). The unbound phosphate was removed by washing the filters extensively in phosphoric acid (0.75%). After removal of the background derived from the control reactions containing the inactive enzyme, the kinase activity data were expressed as the percent of the remaining kinase activity in the test samples compared with the reactions of the vehicle; DMSO. The IC_50_ values and curve fits were obtained using the Graph Pad Prism Software (CA, USA).

### Chemicals and reagents

PTs including CA, AA and UA (Purity > 98% by HPLC), Lipopolysaccharide (LPS from *Escherichia coli* 0111:B4), dimethyl sulfoxide (DMSO), 3-(4,5-dimethylthiazol-2-yl)-2,5-diphenyl tetrazolium bromide (MTT), aprotinin, leupeptin, phenyl methyl sulfonylfluoride (PMSF), dithiothreitol, bovine serum albumin (BSA), Bradford reagent were purchased from Sigma Chemical Co., (St. Louis, MO, USA). Dulbecco’s Modified Eagle’s Medium (DMEM), fetal bovine serum (FBS), penicillin, streptomycin and other reagents for cell culture were from Hi Media (Mumbai, India). All the other chemicals and reagents were purchased from standard sources and were of analytical or HPLC grade, as required. The antibodies specific for Phospho-IKKα/β (Ser176/177), Serine threonine protein kinase (Akt), glycerraldehyde 3-phosphate dehydrogenase (GAPDH) and IFN-γ were purchased from Cell Signaling Technology (CA, USA). The antibodies against NF-κBp65, IKKα, C-Jun and HRP conjugated secondary antibodies were obtained from Santa Cruz Biotechnology (Texas, USA).

### Cell culture and treatments

The RAW 264.7 macrophage cell line was obtained from the American Type Culture Collection (ATCC), Cat# RAW 264.7 ATCC TIB-71 (Manassas, VA, USA). The cells were maintained at 37°C in the DMEM medium supplemented with 10% FBS, penicillin (100 U/ml) and streptomycin sulfate (100 μg/ml) in a humidified atmosphere of CO_2_ (5%). The cells were cultured in culture flasks (75 cm^2^) and the medium was changed every alternate day. When the cultured flasks reached about 70% confluence, the cells were detached by scraping and reseeded into the fresh flasks for continued passage and growth. The cell viability was determined before conducting each experiment [60]. Various concentrations of CA, AA and UA were dissolved in DMSO and added to the medium. For all the experiments involving drug treatment, drugs in the DMSO stocks were added to the cell cultures with the DMSO concentrations maintained below 0.05% v/v [61]. The cells were stimulated with LPS (1 μg/ml) for 20 min following the drug treatment. For treatment, the cells were incubated at various concentrations of CA (20, 50, 70 μM), AA (70, 90, 120 μM) and UA (70, 100, 120 μM).

### Cell viability study (MTT assay)

The cell viability study after drug treatment was performed using MTT [3-(4,5-dimethylthiazol-2yl-)-2,5-diphenyl tetrazolium bromide] viability assay as described earlier [60,62]. Approximately, 10×10^3^ cells were seeded in triplicate in 96-well tissue culture plates. After incubating the cells for 24 h at 37°C in a 5% CO_2_ incubator, the cells were treated with CA, AA and UA at a concentration range from 10 to 150 μM and incubated for further 24 h. After removal of the medium, the cells were washed in a Phosphate- Buffered Saline (PBS) and MTT solutions (0.05 μg/μl) diluted in PBS and added to each well. The plates were incubated at 37°C for 4 h to allow the formation of purple formazan crystals. Thereafter, 100 μl of detergent solution was added to each well to solubilize the crystals and incubated for 30 min at 37°C. The intensity of the formed color was measured spectrophotometrically at 570 nm using a microplate reader (Multimode ELISA reader, Berthold Technologies, Germany). Each data point was executed in triplicate and all assays were performed at least thrice. The data were presented as the percent (%) viability relative to the untreated control.

### Protein expression and western blotting

For quantification of protein expression, western blotting was performed as described earlier [61,63]. The RAW 264.7 cells (1×10^6^) were plated in six-well tissue culture plates, treated with different concentrations of CA, AA and UA for 90 min and stimulated with LPS (1 μg/ml) for 20 min. After treatment, the cells were harvested and the cell lysate was prepared by using a modified RIPA lysis buffer (50 mM tris, 150 mM NaCl, 0.5 mM deoxycholate, 1% NP-40, 0.1% SDS, 1 mM Na_3_VO_4_, 5 mM EDTA, 1 mM PMSF, 2 mM DTT, 10 mM β-glycerophosphate, 50 mM NAF, 0.5% triton X-100, protease inhibitor cocktail). The Bradford’s method was used for the estimation of protein. The protein (60 μg) was loaded and separated in 10% sodium dodecyl sulfate polyacrylamide gel electrophoresis (SDS-PAGE) and transferred to poly vinylidene difluoride (PVDF) membrane. The membrane was blocked either with 5% non-fat milk in Tris buffered saline (TBS) or BSA (5%) in TBS and incubated with antibodies such as rabbit monoclonal antibody against phospho-IKKα (Ser 176)/IKKβ (Ser 177) (Cat # 2078, diluted 1:1000, Cell Signaling Technology, USA), rabbit polyclonal antibody against Akt (Cat # 9272, diluted 1:1000, Cell Signaling Technology, USA), rabbit monoclonal antibody against GAPDH (Cat # 2118, diluted 1:1000, Cell Signaling Technology, USA), rabbit polyclonal NF-κB p65 Antibody (sc-7151, diluted 1:1000, Santa Cruz Biotechnology), rabbit polyclonal c-Jun Antibody (sc-45, diluted 1:1000, Santa Cruz Biotechnology) and rabbit polyclonal IKKα Antibody (sc-7218, diluted 1:1000, Santa Cruz Biotechnology). They were further washed and incubated with goat anti-rabbit IgG-HRP (sc-2004, diluted 1:1000, Santa Cruz Biotechnology). The signals were detected using the ECL western blotting reagent and chemi-luminescence was exposed on Kodak X-Omat films (Sigma, USA).

### Luciferase assay

The transcription factor activity of NF-κB was measured through the Luciferase reporter assay, according to the methods described earlier [62,64]. The RAW 264.7 cells were seeded in six-well plates until achieved 70% confluence and transected by lipofectamine-2000 reagent (Invitrogen, Thermo Fisher Scientific India, Pvt. Ltd., Mumbai, India) following the instructions in manufacturer’s protocol. The NF-κB (2.0 μg) plasmid was used for the transfection and incubated for 6–8 h. The β-gal construct was used and served as an internal control. The NF-κB luciferase constructs p5XIP10 B (containing five tandem copies of the NF-κB site from the IP10 gene) along with the β-gal construct were transfected transiently into the RAW 264.7 cells. After incubation, the media was replaced with fresh media and further incubated for 12 h. The cells were then incubated with varied concentrations of CA, AA and UA for 90 min and exposed to LPS. At the end of incubation, the cells were harvested and washed twice with PBS. The cells were further treated with lysis buffer (NP-40, Protease inhibitor tablet and 100 mM Tris pH 6.8) and the efficiencies of the transfection were normalized by β-galactosidase activity. The luciferase activity was measured using a microplate reader (Tristar, Berthold Technologies, Germany).

### Indirect ELISA for detection of IFN-γ

The *in vitro* detection of soluble IFN-γ in the cell culture supernatant was performed by the indirect ELISA method, according to the protocol mentioned earlier [65]. Briefly, the protein antigen was mixed in a 100 μl/well of the coupling buffer and then coated onto the 96-well microplate (Corning, Cat #3679). After being incubated overnight at 4°C, the wells were washed twice with 1X PBS containing Tween 20 (0.1% v/v)/well followed by a blocking of the residual binding sites with a super-cocktail buffer (1% w/v BSA, 1% w/v ovalbumin and 0.1% Tween-20 in 1X PBS). Next, diluted primary anti-IFN-γ antibody was added and the plate was incubated for 2 h at room temperature. After washing with fresh 1X PBST, the wells were incubated with HRP-conjugated anti-mouse antibody for 1 h, followed by thrice washing with 1X PBST. The freshly prepared 2, 2’-azinobis-(3-ethylbenzthiazoline-6-sulphonic acid) substrate solution (0.5 mg/ml) in the citrate buffer was added and incubated at room temperature in dark for 10 min. The absorbance of the colored product formed was detected at 450 nm using the microplate ELISA reader (BioTek Instruments, USA).

### Statistical analysis

The statistical analysis was performed using the Graphpad Prism software (CA, USA). A one-way analysis of variance (ANOVA) followed by Dunnett test was used for the statistical comparisons of the groups unless otherwise indicated. The differences were considered statistically significant when P < 0.05.

## Results

### PASS predicted majority of PTs as NF-κB inhibitors

The virtual screening protocol used in this study is based on the sequential application of filters to explore the selective IKKβ mediated NF-κB inhibitory PTs. The workflow was as shown in S1 Fig. The virtual screening resulted in the establishment of a compound library containing 109 prospective NF-κB inhibitory PTs. This manually assembled dataset of PTs was screened through the PASS software to identify the potential NF-κB inhibitors based on the PASS scores. The PASS predicts the probable biological potential of the chemicals based on structural formula and offers the predicted activities as the probability of activity (Pa) and inactivity (Pi) [66–68]. The higher Pa value indicates less probability of obtaining the false-positive results of the PASS prediction during the course of biological testing [69]. Out of 109 molecules screened by PASS, 80 molecules exhibit ability to inhibit the NF-κB with an activity score (Pa) greater than 0.3 (Table 1). These 80 PTs with predicted NF-κB inhibitory activity were subjected to further docking study.

**Table 1:** PASS predicted activity scores of pentacyclic triterpenoids for NF-κB inhibitor activity.

Sr. No	Compound	Predicted score for Transcription factor NF-kappa B inhibitor activity	
		Pa	Pi
**1**	**Boswellic acid 2e**	**0,735**	**0,002**
**2**	**Boswellic acid 2c**	**0,728**	**0,002**
**3**	**Boswellic acid 2d**	**0,717**	**0,002**
**4**	**Boswellic acid 2a**	**0,691**	**0,003**
**5**	**Rehmannic acid**	**0,677**	**0,003**
**6**	**3b-trans-feruloyloxy-16b-hydroxylup-20(29)-ene**	**0,677**	**0,003**
**7**	**Boswellic acid 2b**	**0,676**	**0,003**
**8**	**Boswellic acid 2f**	**0,667**	**0,003**
**9**	**Glycyrrhetic acid**	**0,659**	**0,003**
**10**	**Sumaresinolic acid**	**0,656**	**0,003**
**11**	**Maniladiol**	**0,656**	**0,003**
**12**	**Boswellic acid 2g**	**0,648**	**0,003**
**13**	**Beta amyrin**	**0,648**	**0,003**
**14**	**Glycyrrhizin**	**0,641**	**0,003**
**15**	**Ursolic acid**	**0,638**	**0,003**
**16**	**Taraxerol acetate**	**0,635**	**0,003**
**17**	**Gymnemic acid I**	**0,633**	**0,003**
**18**	**Katonic acid**	**0,626**	**0,003**
**19**	**Imberbic acid**	**0,623**	**0,003**
**20**	**Oleanolic acid**	**0,620**	**0,003**
**21**	**3beta—acetoxy beta—amyrin**	**0,620**	**0,003**
**22**	**Coussaric acid**	**0,612**	**0,003**
**23**	**3 beta acetoxy alfa amyrin**	**0,607**	**0,003**
**24**	**Taraxasterol acetate**	**0,605**	**0,003**
**25**	**Alphitolic acid**	**0,603**	**0,004**
**26**	**Corosolic acid**	**0,597**	**0,004**
**27**	**Hederagenin**	**0,578**	**0,004**
**28**	**Madecassic acid**	**0,577**	**0,004**
**29**	**Augustic acid**	**0,577**	**0,004**
**30**	**2a,3a,19-trihydroxy-olean-12-en-23,28-dioic acid**	**0,568**	**0,004**
**31**	**2a,3a, 24-trihydroxyurs-12, 20(30)-dien-28-oic acid**	**0,568**	**0,004**
**32**	**Uvaol**	**0,567**	**0,004**
**33**	**Betunaldehyde**	**0,552**	**0,004**
**34**	**3b-trans-sinapoyloxylup-20(29)-en-28-ol**	**0,549**	**0,004**
**35**	**Asiatic acid**	**0,547**	**0,004**
**36**	**Emarginellic acid**	**0,545**	**0,004**
**37**	**3-O-acetylbetulinic acid**	**0,544**	**0,004**
**38**	**Polygalacic acid**	**0,541**	**0,004**
**39**	**Erythrodiol**	**0,541**	**0,004**
**40**	**Ursonic acid**	**0,540**	**0,004**
**41**	**Acetylursolic acid**	**0,539**	**0,004**
**42**	**Morolic acid**	**0,535**	**0,004**
**43**	**Arnidiol**	**0,532**	**0,004**
**44**	**Bartogenic acid**	**0,523**	**0,004**
**45**	**Barrinic acid**	**0,523**	**0,004**
**46**	**Barrigenic acid**	**0,523**	**0,004**
**47**	**19 epibartogenic acid**	**0,523**	**0,004**
**48**	**Hyptatic acid A**	**0,521**	**0,004**
**49**	**Bayogenin**	**0,521**	**0,004**
**50**	**Tanginol**	**0,512**	**0,004**
**51**	**Lupeol**	**0,511**	**0,004**
**52**	**CDDO methyl ester**	**0,502**	**0,005**
**53**	**Bryonolic acid**	**0,501**	**0,005**
**54**	**20-Epibryonolic acid**	**0,501**	**0,005**
**55**	**Germanicol**	**0,495**	**0,005**
**56**	**Faradiol**	**0,480**	**0,005**
**57**	**Crotalic acid**	**0,478**	**0,005**
**58**	**Betulinic acid**	**0,464**	**0,005**
**59**	**23-epoxy-friedelan-28-oic acid**	**0,460**	**0,005**
**60**	**Arjunoic acid**	**0,451**	**0,005**
**61**	**Pomolic acid**	**0,432**	**0,006**
**62**	**Impressic acid**	**0,427**	**0,006**
**63**	**Tangulic acid**	**0,422**	**0,006**
**64**	**Madecassoside**	**0,410**	**0,006**
**65**	**Canophyllal**	**0,403**	**0,007**
**66**	**Heliantriol C**	**0,395**	**0,007**
**67**	**CDDO**	**0,386**	**0,007**
**68**	**Anhydrobartogenic acid**	**0,384**	**0,007**
**69**	**Acutangulic acid**	**0,371**	**0,008**
**70**	**Asiaticoside**	**0,366**	**0,008**
**71**	**Phytolaccagenin**	**0,365**	**0,008**
**72**	**Amooranin**	**0,363**	**0,008**
**73**	**Rotundic acid**	**0,354**	**0,009**
**74**	**Friedeline**	**0,353**	**0,009**
**75**	**Euscaphic Acid (Tormantic acid)**	**0,352**	**0,009**
**76**	**Lupeol acetate**	**0,352**	**0,009**
**77**	**Epifriedelanol**	**0,339**	**0,009**
**78**	**Betulin**	**0,334**	**0,010**
**79**	**Tiarellic acid**	**0,311**	**0,011**
**80**	**Heliantriol B**	**0,308**	**0,011**
*81*	*Betulonic acid*	*0*,*298*	*0*,*012*
*82*	*3-oxo-friedelan-28-oic-acid*	*0*,*295*	*0*,*012*
*83*	*2a*,*3ß*,*19a*,*23-tetrahydroxyurs-12-en-28-oic acid*	*0*,*293*	*0*,*012*
*84*	*Canophyllic acid*	*0*,*287*	*0*,*013*
*85*	*Zizyberanalic acid*	*0*,*286*	*0*,*013*
*86*	*Moronic acid*	*0*,*275*	*0*,*014*
*87*	*Rhoiptelenone*	*0*,*252*	*0*,*016*
*88*	*CDDO analoge 2-cyano-3*,*12-dioxooleana-1*,*9(11)-dien-28-onitrile*	*0*,*247*	*0*,*017*
*89*	*Glycyrrhetic acid 6c*	*0*,*242*	*0*,*018*
*90*	*24 hydroxy tormantic acid*	*0*,*240*	*0*,*018*
*91*	*Celastrol/ Tripterin*	*0*,*222*	*0*,*021*
*92*	*Goreishic acid I*	*0*,*219*	*0*,*022*
*93*	*Betulinic acid derivative 1 f*	*0*,*219*	*0*,*022*
*94*	*Zizyberenalic acid*	*0*,*215*	*0*,*023*
*95*	*Betulinic acid derivative 1 g*	*0*,*214*	*0*,*023*
*96*	*Canophyllol*	*0*,*212*	*0*,*024*
*97*	*2-amino-3-hydroxy-2-(hydroxymethyl) propyl betulonate*	*0*,*194*	*0*,*029*
*98*	*Ceanothic acid*	*0*,*184*	*0*,*032*
*99*	*Prisimerin*	*0*,*181*	*0*,*033*
*100*	*Pinfaenoic acid*	*0*,*180*	*0*,*034*
*101*	*24-hydroxybutulinic acid*	*0*,*165*	*0*,*039*
*102*	*Tingenone*	*0*,*155*	*0*,*044*
*103*	*CDDO-imidazolide*	*0*,*152*	*0*,*045*
*104*	*Goreishic acid II*	*0*,*152*	*0*,*045*
*105*	*N-(2*,*3-hydroxy-2-(hydroxymethyl) propyl) (3-O-acetyl) betulinamide*	*0*,*146*	*0*,*048*
*106*	*3-oxa-24-hydroxybutulinic acid*	*0*,*134*	*0*,*057*
*107*	*Tingenin*	*0*,*128*	*0*,*062*
*108*	*N-(2*,*3-hydroxy-2-(hydroxymethyl) propyl) betulinamide*	*0*,*112*	*0*,*081*
*109*	*Beta sitosterol*	*0*,*100*	*0*,*098*

Compounds with Pa > 0.3 are highlighted in bold; compounds with Pa < 0.3 are highlighted in italic

### PTs dock on NEMO/IKKβ association complex where it binds to a steroidal lactone, Withaferin A

The molecular model of the IKKβ for virtual screening was built using the x-ray co-crystal structure of NEMO/IKKβ association domain (PDB: 3BRV). A study by Grover et al. [10] reported the docking of Withaferin A, a constituent of *Withania somnifera* root on the crystal structure of the NEMO/IKKβ protein. Withaferin A belongs to a class of naturally occurring C-28 steroidal lactones possessing an ergostane skeleton. The PTs have structural features resembling steroidal compounds like Withaferin A, which bind to the NEMO/IKKβ. Hence, we docked our compound library onto the NEMO/IKKβ association complex to explore the binding modes of the PTs with this complex and to identify the IKKβ inhibitors.

The 80 PTs obtained from the first filter of PASS program were subjected to a docking study on the binding pocket of the IKKβ by the XP docking mode of Schrodinger. The docking results showed that only 56 ligands had a glide score (docking score) less than -1.50 as presented in Table 2.

**Table 2:** Docking results of PTs on IKKβ based on glide dock score and number of hydrogen bond interactions (Schrodinger 9.0).

Sr. No.	Compound	Dock score	Number of hydrogen bonds
**1**	**Madecassoside**	**-8.7101**	**8**
**2**	**Asiaticoside**	**-7.7322**	**6**
**3**	**Glycyrrhizin**	**-5.7329**	**2**
**4**	**Gymnemic acid I**	**-5.5447**	**4**
**5**	**Tangulic acid**	**-4.8562**	**3**
**6**	**2a,3a,19-trihydroxy-olean-12-en-23,28-dioic acid**	**-4.2956**	**4**
**7**	**2a,3a,24-trihydroxyurs-12,20(30)-dien-28-oic acid**	**-4.2930**	**2**
**8**	**Bartogenic acid**	**-4.0445**	**5**
**9**	**19 epibartogenic acid**	**-3.9578**	**3**
**10**	**Corosolic acid**	**-3.7280**	**2**
**11**	**Tanginol**	**-3.6536**	**4**
**12**	**Arjunoic acid**	**-3.5731**	**3**
**13**	**Anhydrobartogenic acid**	**-3.5693**	**1**
**14**	**Tiarellic acid**	**-3.4499**	**2**
**15**	**Boswellic acid 2c**	**-3.3045**	**1**
**16**	**Madeccassic acid**	**-3.2643**	**2**
**17**	**Boswellic acid 2a**	**-3.2525**	**1**
**18**	**Katonic acid**	**-3.2434**	**3**
**19**	**Bayogenin**	**-3.1951**	**1**
**20**	**Boswellic acid 2e**	**-3.1793**	**2**
**21**	**Emarginellic acid**	**-3.1409**	**1**
**22**	**Asiatic acid**	**-3.1083**	**1**
**23**	**Hederagenin**	**-3.0852**	**0**
**24**	**Hyptatic acid A**	**-3.0150**	**2**
**25**	**3b-trans-sinapoyloxylup-20(29)-en-28-ol**	**-3.0140**	**2**
**26**	**Phytolaccagenin**	**-3.0097**	**2**
**27**	**Rehmannic acid**	**-2.8629**	**1**
**28**	**Polygalacic acid**	**-2.8240**	**2**
**29**	**Bryonolic acid**	**-2.8235**	**2**
**30**	**3b-trans-feruloyloxy-16b-hydroxylup-20(29)-ene**	**-2.6539**	**2**
**31**	**Morolic acid**	**-2.6240**	**2**
**32**	**Acutangulic acid**	**-2.6235**	**1**
**33**	**Glycyrrhetic acid**	**-2.5019**	**2**
**34**	**3-O-acetylbetulinic acid**	**-2.4949**	**2**
**35**	**Alphitolic acid**	**-2.4262**	**1**
**36**	**Uvaol**	**-2.4097**	**1**
**37**	**Coussaric acid**	**-2.3997**	**1**
**38**	**Euscaphic acid (Tormantic acid)**	**-2.3726**	**3**
**39**	**Barrinic acid**	**-2.3327**	**2**
**40**	**23-epoxy-friedelan-28-oic acid**	**-2.3364**	**1**
**41**	**Boswellic Acid 2d**	**-2.3118**	**0**
**42**	**Barrigenic acid**	**-2.2739**	**3**
**43**	**Imberbic acid**	**-2.1774**	**2**
**44**	**Sumaresinolic acid**	**-2.1574**	**3**
**45**	**Oleanolic acid**	**-2.0575**	**0**
**46**	**Betulinic acid**	**-1.9764**	**2**
**47**	**Rotundic acid**	**-1.9701**	**2**
**48**	**Amooranin**	**-1.9671**	**1**
**49**	**3 beta acetoxy alfa amyrin**	**-1.8498**	**1**
**50**	**Faradiol**	**-1.8395**	**1**
**51**	**Erythrodiol**	**-1.7976**	**2**
**52**	**Crotalic acid**	**-1.7286**	**1**
**53**	**Ursolic acid**	**-1.6268**	**1**
**54**	**Germanicol**	**-1.5808**	**1**
**55**	**Betulin**	**-1.5778**	**2**
**56**	**Pomolic acid**	**-1.5199**	**1**
*57*	*Augustic acid*	*-1*.*4956*	*2*
*58*	*Lupeol*	*-1*.*4399*	*0*
*59*	*Taraxasterol acetate*	*-1*.*4049*	*1*
*60*	*Taraxerol acetate*	*-1*.*3923*	*1*
*61*	*3beta—acetoxy beta—amyrin*	*-1*.*3176*	*1*
*62*	*Arnidiol*	*-1*.*3012*	*1*
*63*	*CDDO*	*-1*.*2843*	*1*
*64*	*Betunaldehyde*	*-1*.*2311*	*2*
*65*	*Boswellic acid 2b*	*-1*.*1577*	*2*
*66*	*Maniladiol*	*-1*.*1569*	*0*
*67*	*Acetylursolic acid*	*-1*.*1135*	*2*
*68*	*Boswellic acid 2f*	*-0*.*9649*	*0*
*69*	*Epifriedelanol*	*-0*.*9104*	*1*
*70*	*20-Epibryonolic acid*	*-0*.*8201*	*2*
*71*	*Heliantriol C*	*-0*.*7831*	*0*
*72*	*Canophyllal*	*-0*.*6019*	*0*
*73*	*Beta amyrin*	*-0*.*4170*	*1*
*74*	*Friedeline*	*-0*.*3956*	*1*
*75*	*Lupeol acetate*	*-0*.*3760*	*0*
*76*	*Ursonic acid*	*-0*.*3209*	*0*
*77*	*Heliantriol B*	*-0*.*2398*	*1*
*78*	*CDDO methyl ester*	*-0*.*1745*	*1*
*79*	*Impressic acid*	*0*.*0737*	*3*
*80*	*Boswellic acid 2g*	*0*.*5091*	*0*

Compounds that pass the docking filter are highlighted in bold; compounds that do not pass the docking filter are highlighted in italic

**Fig 1:**
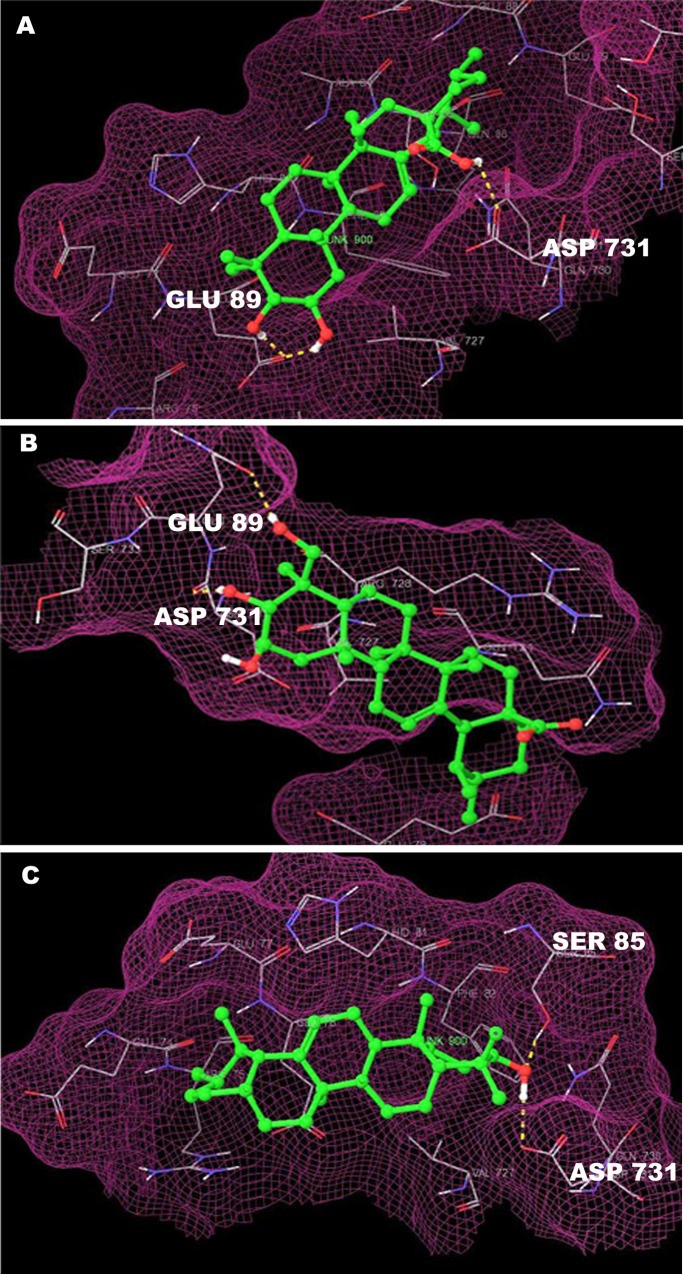
Hydrogen bond interaction of CA (A), AA (B) and UA (C) at GLU 89 and SER 85 gatekeeper residues of NEMO/IKKβ association complex (PDB code: 3BRV).

The compounds having scores greater than the cutoff score of -1.50 were not considered for further study. The residues 85–101 of dimeric NEMO formed a flat slit, paving the way to two broad and extensive IKK-binding pockets, each pocket being occupied by the IKK peptide linker and the NEMO binding domain (NBD). The IKK peptide formed intermolecular hydrogen-bond interactions (SER 85 and GLU 89) with NEMO in the NEMO specificity pocket. Three large IKK side chains inside the NEMO pocket, which form the consolidated intermolecular hydrophobic interactions (LEU 93, PHE 92, MET 94, PHE 97, ALA 100, and ARG 101) are responsible for the formation of the NEMO-IKKβ complex [10]. The PTs studied herein fit in the same pocket and interact with the SER 85 and GLU 89 amino acids (Fig 1). Apparently, the CA and AA interacted with the GLU 89 whereas, the UA interacted with the SER 85. Further, all these three molecules showed interaction with the ASP 731. Among the three PTs, CA, AA and UA were selected for further extensive *in vitro* testing.

### PTs abide by the softened Lipinski’s Rule of Five and show a favorable pharmacokinetic profile

The total 56 PTs obtained from the second filter of docking were further screened through the next filter of Lipinski’s Rule of Five to reinforce the process of identifying the drug likeliness of the selected IKKβ inhibitory PTs. Natural products including the isolated phytochemicals generally violate the Lipinski’s Rule of Five [49,56,57]. To prevent the possibility of missing prospective compounds from this class of PTs, we applied the softened Rule of Five and a violation of one of the Lipinski’s Rule of Five was within considerable limits. Among the 56 molecules screened through this filter, 45 molecules were in compliance with the softened Lipinski’s Rule of Five. Based on the availability from the reliable commercial sources, three PTs, namely CA, AA and UA containing distinctive chemical scaffolds were chosen for further study.

The number of violations of the Lipinski’s Rule of Five for AA is zero, although CA and UA violate only one. The CA, AA and UA have molecular weight less than 500 Da and the number of HBD is less than 5 and HBA is less than 10. The predicted octanol/water partition coefficient (QPlogPo/w), is critical for the estimation of the absorption and distribution of drugs within the body. It was found slightly higher than 5 for CA (5.20) and UA (6.15), although it falls within the acceptable range for AA (4.08). The aqueous solubility (log S) for only UA was above the recommended limits of -6.5 to 0.5. The predicted IC_50_ value for the blockage of the HERGK+ channels (QP logHERG) for CA, AA and UA was within the acceptable range (i.e. > - 5.0). The predicted cell permeability (QPPCaco), a factor responsible for the drug metabolism and its access to the biological membrane, lies within the suitable range of 25 to 500 for CA, AA and UA. The predicted value of binding to human serum albumin (QPlogkhsa) for three triterpenoids was within the acceptable limit, -1.5 to 1.5. When the suitable route of drug administration is oral, drug absorption is the main concern. The percentage of the oral absorption for three triterpenoids was not only within the acceptable range (25%: poor and > 80%: high) but also at its highest limits, indicating better predicted absorption of these triterpenoids through the oral routes of administrations (S2 Table). Thus, CA and UA follow the softened Lipinski’s Rule of Five, while AA follows the classical Lipinski’s Rule of Five. The majority of the pharmacokinetic parameters of the PTs were within the acceptable range defined for human use (see S2 Table footnote), thereby indicating their potential as drug-like molecules.

### PTs inhibited human IKKβ in cell free kinase assay

**Fig 2:**
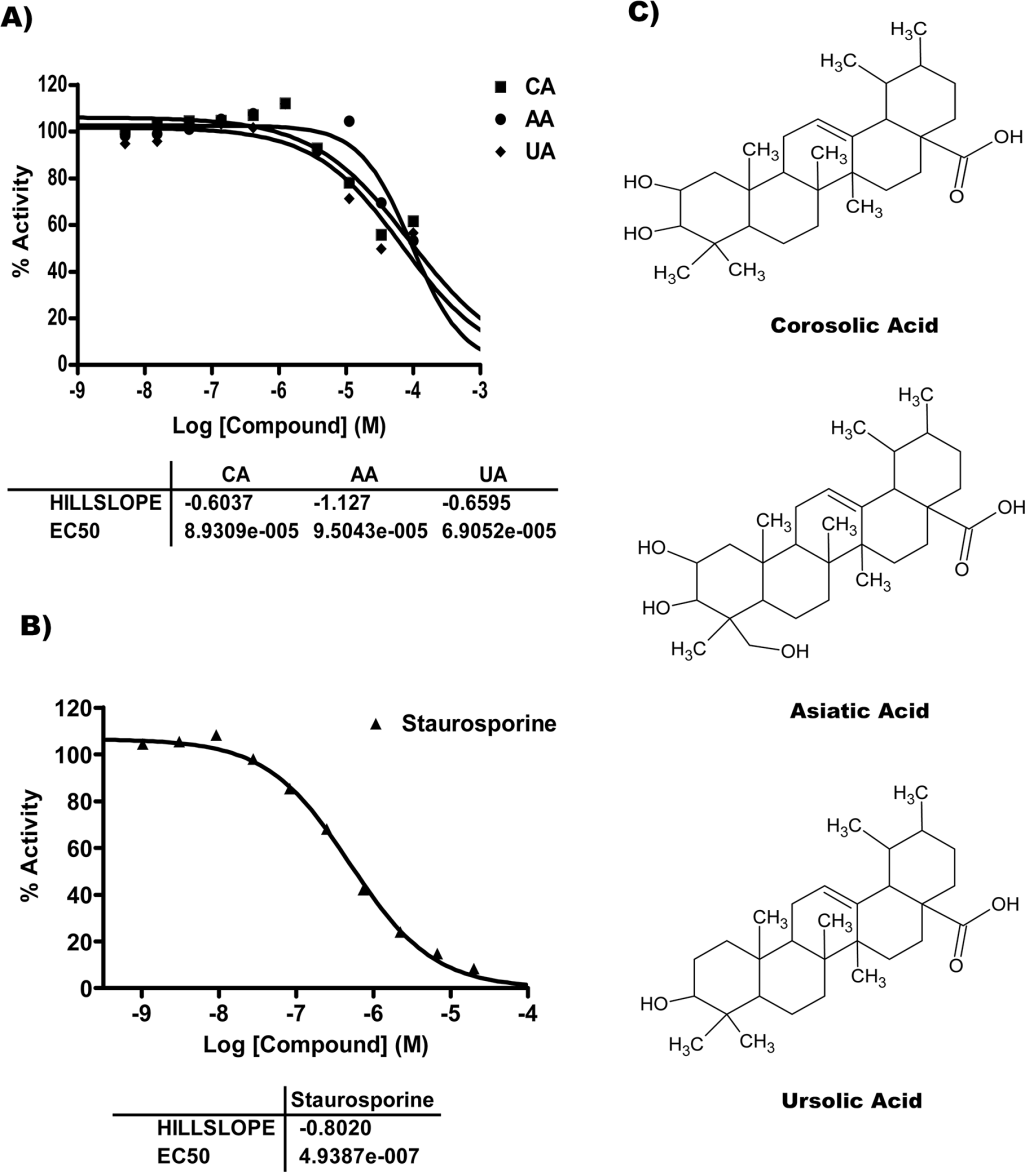
CA, AA and UA inhibited *in vitro* IKKβ kinase activity. Dose response results of *in vitro* kinase assay for three compounds CA (■), AA (●) and UA (♦) against IKKβ. A) IC_50_ data of compounds against IKKβ. B) IC_50_ data of standard staurosporine against IKKβ. C) Chemical structures of CA, AA and UA. Data point represents averages of two independent replicates. Data exhibiting inhibition was fitted with a sigmoidal dose-response curve to derive IC_50_ values.

To confirm the results of the molecular docking studies and to check whether the inhibition of the NF-κB is mediated through the inhibition of the IKKβ, we performed HotSpot kinase assay against the human IKKβ. The IC_50_ values for the IKKβ inhibition for CA, AA and UA were 89.3 μM, 95.0 μM and 69.0 μM, respectively, when compared with 493.9 nM for staurosporine. Hence, CA, AA and UA demonstrated their ability to act against human IKKβ. The UA was found to have better ability to inhibit phosphorylation of the human IKKβ as compared with CA and AA in the cell free assay (Fig 2).

### CA, AA and UA affect the viability of RAW 264.7 cells

**Fig 3:**
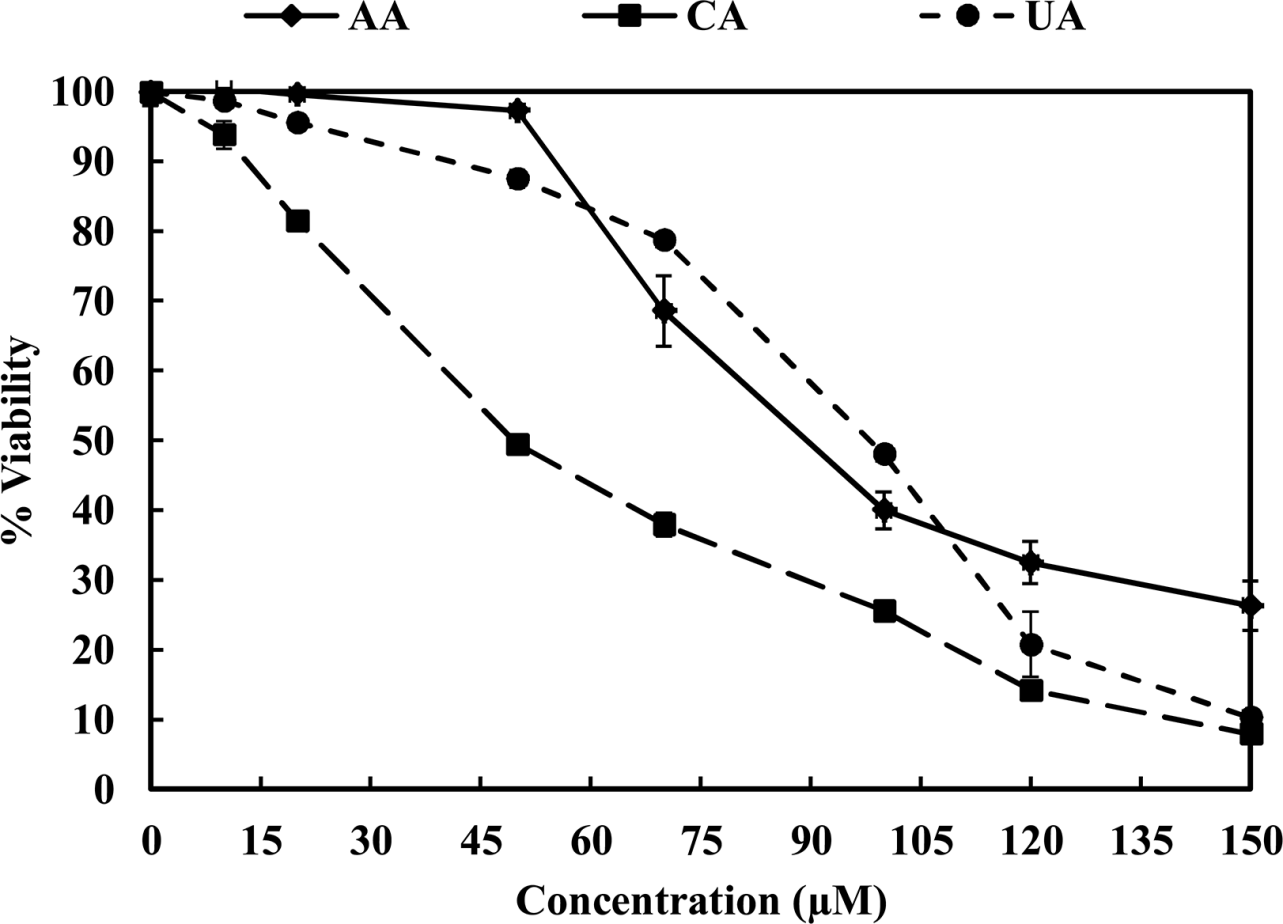
CA, AA and UA affect viability of RAW 264.7 cells as evident in MTT assay. Cytotoxicity was evaluated by MTT assay after exposure of RAW 264.7 cells to increasing concentrations of CA (■), AA (♦) and UA (●) (10 to 150 μM) for 24 h. Cells were plated in each well (10,000 cells/well) of the 96-well tissue-culture plates, after 70% confluence, cells were treated with different concentrations of CA (■), AA (♦) and UA (●) for 24 h. After drug treatment MTT assay was performed as described under materials and methods section. Data were plotted as percent viability (% control).

The cytotoxicity of the PTs was determined by MTT assay before evaluating their effects on the IKK/NF-κB signaling. The cytotoxicity of the chosen triterpenoids was measured in the presence of the LPS by using the MTT in the RAW 264.7 cells. The RAW 264.7 cells were then exposed to increasing doses of CA, AA and UA. The 50% cell growth inhibitory concentration (IC_50_) obtained for CA, AA and UA in the macrophages were 50 μM, 90 μM and 100 μM, respectively (Fig 3).

The MTT assays revealed that pretreatment of the macrophages with these molecules alters the growth of the cells. The molecules were tested at IC_50_ concentrations in further experiments. To exclude the possibility that the cytotoxic effects of these compounds could be attributed to their effects on the NF-κB cascade, the PTs were used at concentrations which were devoid of any non specific cytotoxic effect.

### PTs inhibited the LPS mediated inflammation through the Akt-IKKα/β-NF-κB pathway in the RAW 264.7 cells

**Fig 4:**
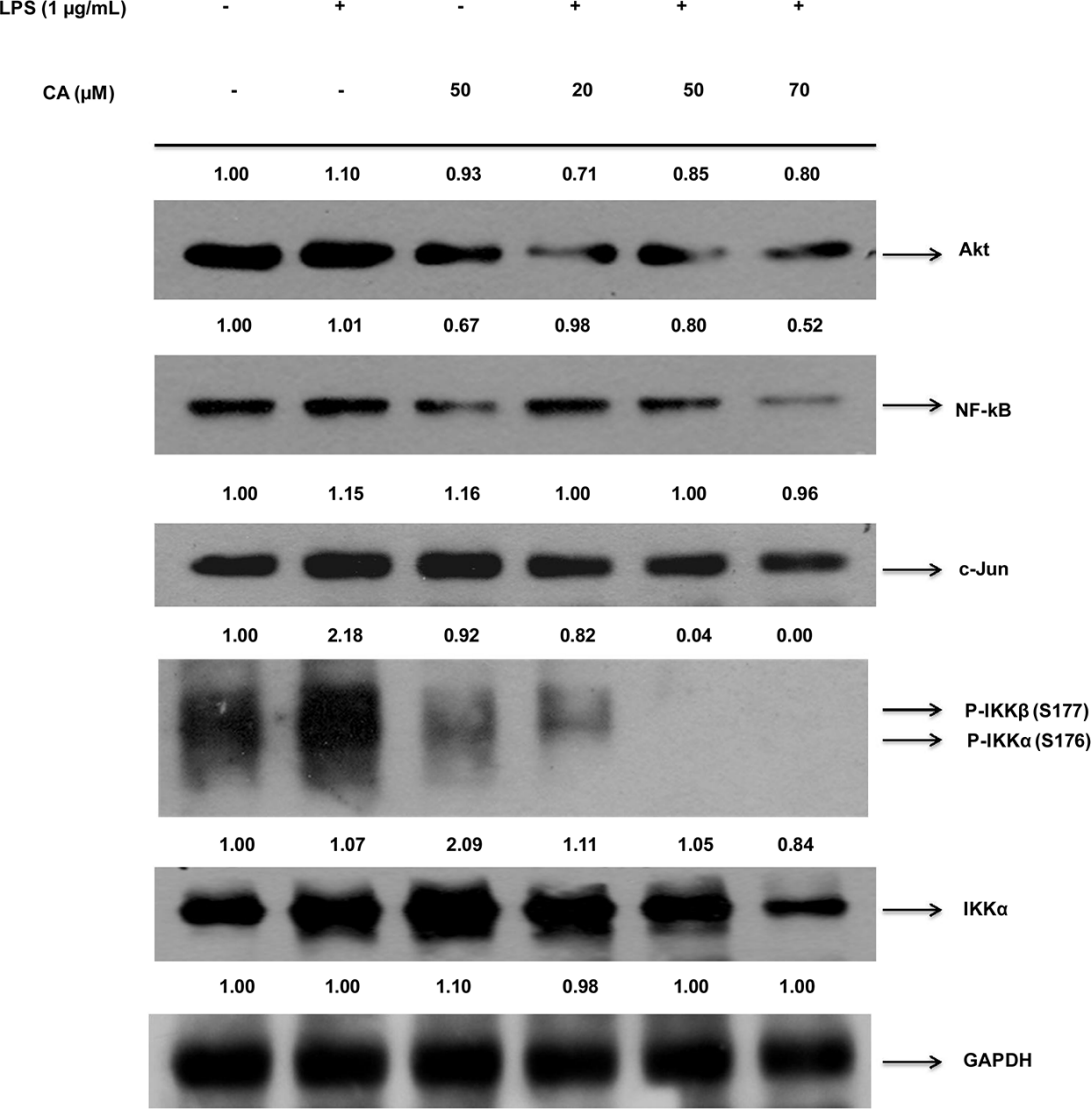
CA inhibited Akt-IKKα/β-NF-κB pathway in RAW 264.7 cells. Cells were pre-treated for 90 min with different concentrations of CA (20, 50, 70 μM) and then treated with LPS (1 μg/ml) for 20 min. Nuclear extracts were prepared as described in materials and methods section and determined for western blotting of NF-κB and Phospho-IKKα/β proteins using specific antibodies. GAPDH served as both an internal loading control and as a nuclear fractionation control. Density ratios versus GAPDH were determined by densitometry. Results are representative of three independent experiments.

**Fig 5:**
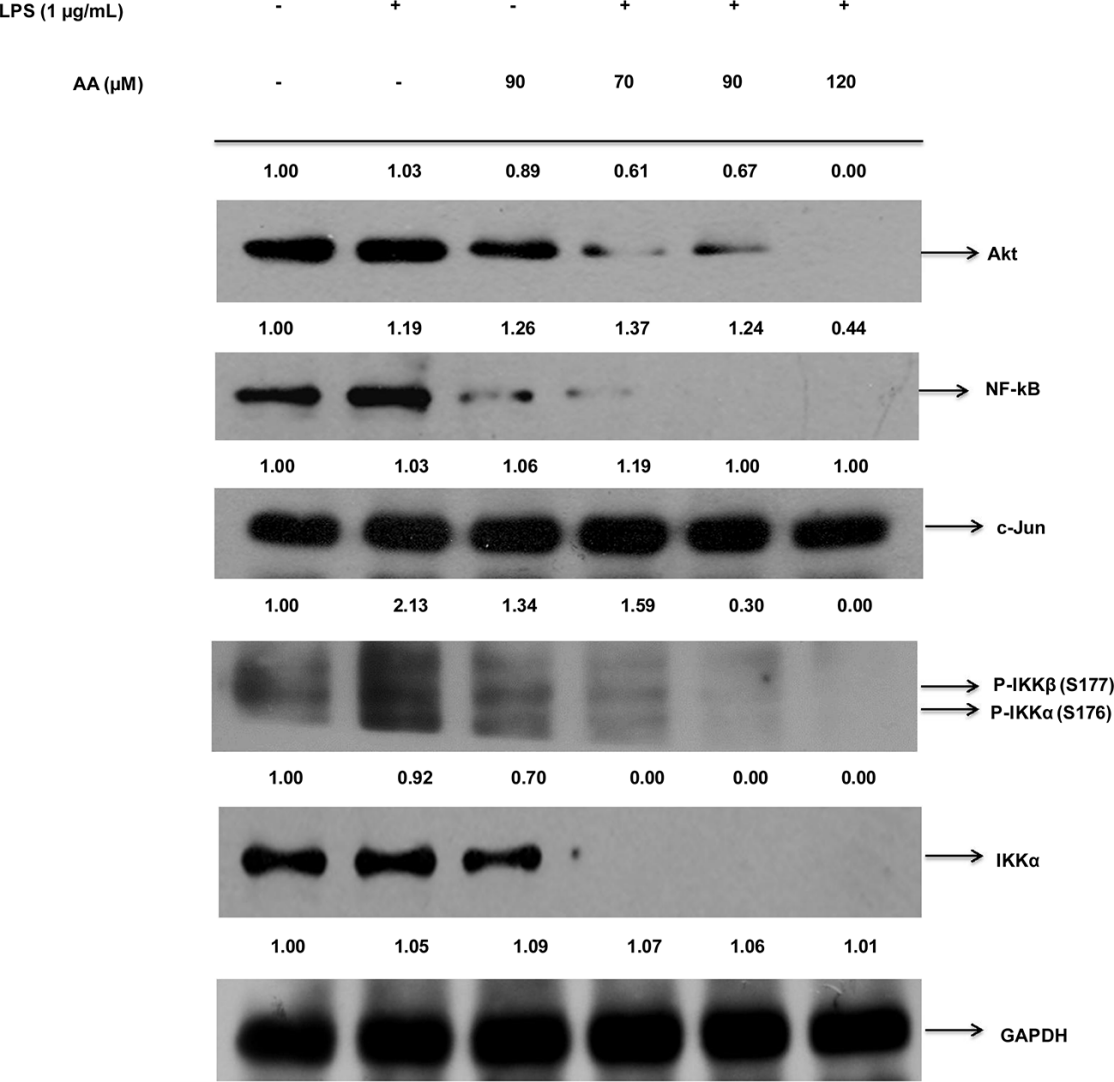
AA inhibited Akt-IKKα/β-NF-κB pathway in RAW 264.7 cells. Cells were pre-treated for 90 min with different concentrations of AA (70, 90,120 μM) and then treated with LPS (1 μg/ml) for 20 min. Nuclear extracts were prepared as described in materials and methods section and determined for western blotting of NF-κB and Phospho-IKKα/β proteins using specific antibodies. GAPDH served as both an internal loading control and as a nuclear fractionation control. Density ratios versus GAPDH were determined by densitometry. Results are representative of three independent experiments.

**Fig 6:**
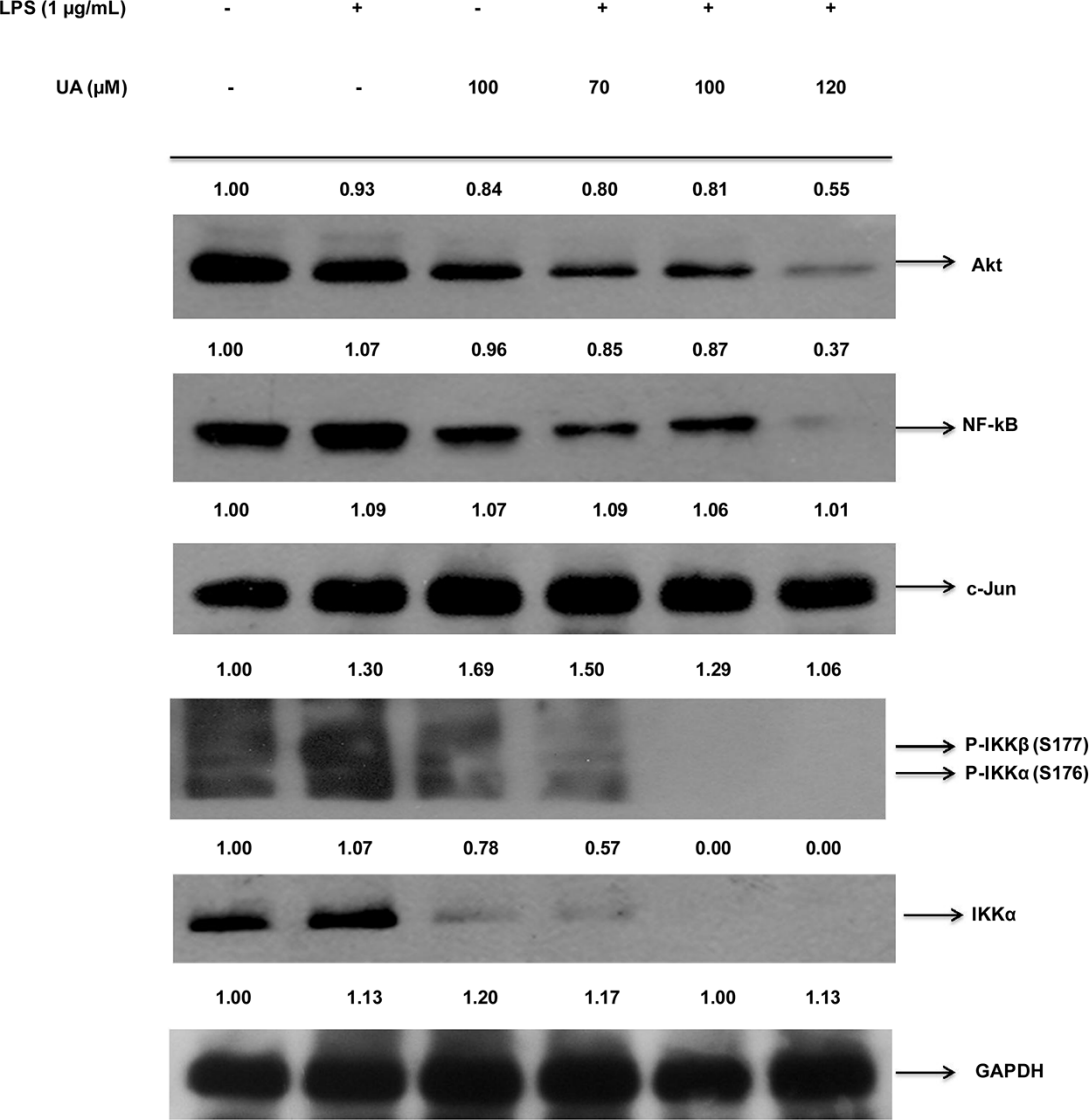
UA inhibited Akt-IKKα/β-NF-κB pathway in RAW 264.7 cells. Cells were pre-treated for 90 min with different concentrations of UA (70,100, 120 μM) and then treated with LPS (1 μg/ml) for 20 min. Nuclear extracts were prepared as described in materials and methods section and determined for western blotting of NF-κB and Phospho-IKKα/β proteins using specific antibodies. GAPDH served as both an internal loading control and as a nuclear fractionation control. Density ratios versus GAPDH were determined by densitometry. Results are representative of three independent experiments.

After the LPS challenge of the RAW 264.7 cells, the preliminary signal leading to the degradation of the IκBα and the activation of the NF-κB is the IKK-dependent phosphorylation of the IκB. The CA, AA and UA markedly inhibited the IKKα/β phosphorylation. In an attempt to investigate the effect of the PTs on the NF-κB and to test whether the inhibition of the NF-κB by the PTs is IKKβ dependent, we also examined the NF-κB and phosphorylated IKKα/β protein expressions in the LPS challenged RAW 264.7 cells. After treatment with the LPS (1 μg/ml) alone for 20 min, the phosphorylated IKKα/β levels were markedly increased and the pretreatment with CA, AA and UA for 90 min before the LPS stimulation reduced the LPS-induced IKKβ phosphorylation and the NF-κB expression. The CA inhibited the IKKα/β phosphorylation and the subsequent NF-κB expression in a concentration dependent manner at 20, 50, 70 μM (Fig 4). The AA and UA also demonstrated the dose-dependent inhibition of the IKKα/β phosphorylation. However, the observed effects of AA and UA were at a concentration higher than that of the CA (Figs 5 and 6). The expression of NF-κB was also decreased by the AA and UA.

Besides the IKK, other factors like Akt and C-Jun are regulators of the inflammatory mediators and NF-κB activation. Therefore, we further determined the extent to which the Akt and C-Jun levels were altered by the PTs tested. The CA, AA and UA suppressed the expression of the Akt (Figs 4, 5 and 6) in addition to the IKKα/β and NF-κB. Interestingly, a meager change in the expression of the c-Jun was observed in the compound treated samples. The C-Jun regulates the cell cycle progression and apoptosis by distinct mechanisms and is a good marker of cell apoptosis [70]. The results indicated that the anti-inflammatory effects of the CA, AA and UA are mediated initially by their action on the LPS-induced inflammatory pathway at concentrations which do not induce apoptosis. However, the degree of protein inhibition involved in the NF-κB activation is different for the triterpenoids tested, with the maximum inhibitory effect being exerted by the CA at concentrations lower than that of the AA and UA. Thus CA, AA and UA caused a discernible blockade of LPS-induced activation of the NF-κB through the inhibition of IKKβ phosphorylation (Figs 4, 5 and 6).

**Fig 7:**
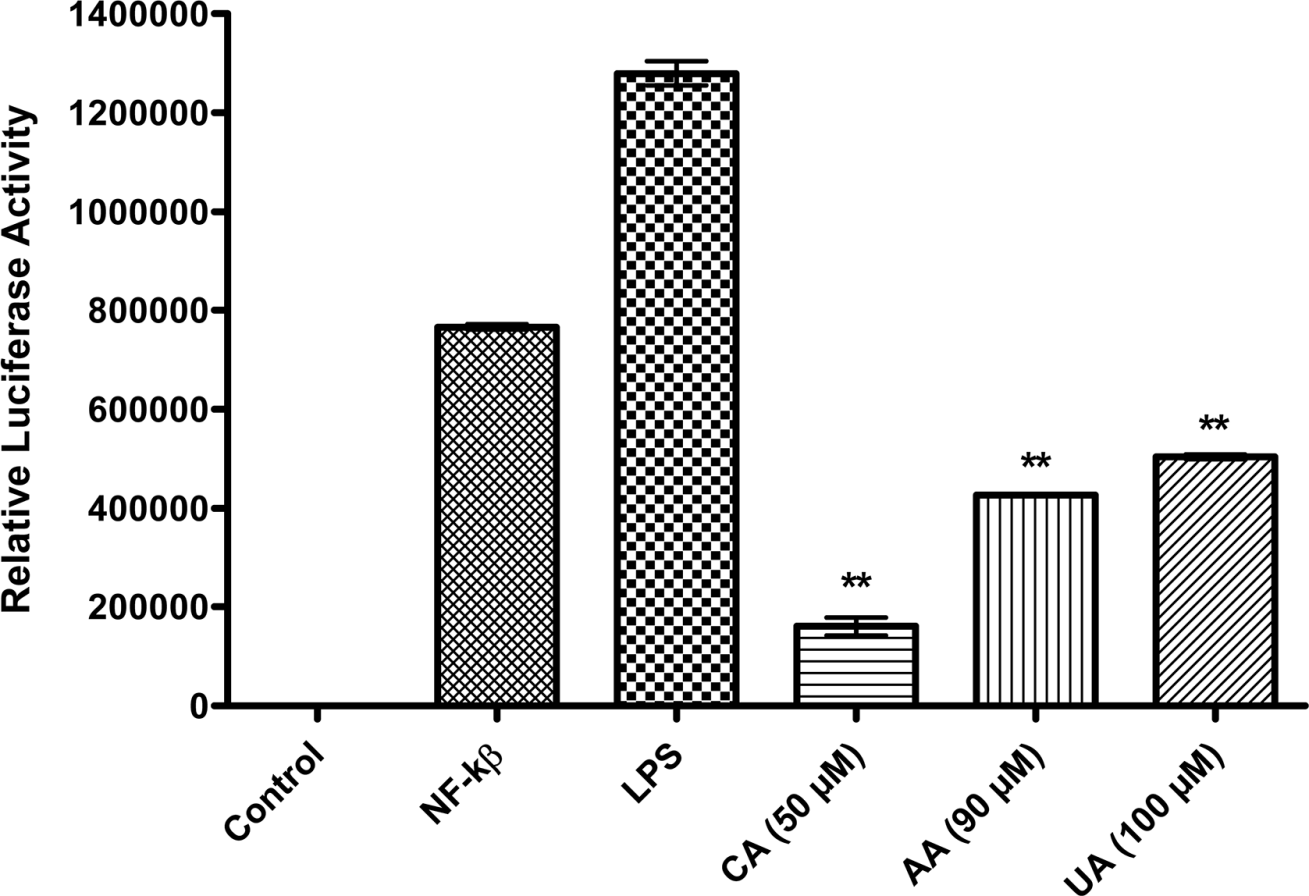
Effects of CA, AA and UA on LPS-induced NF-κB transcriptional activity. Cells were transiently co-transfected with pNF-κB-luc reporter and then either left untreated (Control) or were pre-treated with IC_50_ concentrations of CA (50 μM), AA (90 μM) and UA (100 μM) for 90 min. LPS (1 μg/ml) was added and cells were further incubated for 4 h, then harvested and luciferase activities were determined using a luciferase assay system and a luminometer. Bars show means ± SD (n = 3). *P (ANOVA) < 0.05 versus LPS only-treated group.

The NF-κB controls the expression of the proteins which are involved in the inflammatory process during pathogenesis of chronic diseases [71–73]. To observe whether the PTs diminish the LPS-induced NF-κB transcriptional activity, we used the luciferase reporter assay. The RAW 264.7 cells were transiently transfected with the NF-κB-dependent luciferase reporter plasmid (pNF-κB-luc). The cells were treated with the LPS (1 μg/ml) alone or co-treated with LPS and PTs, as described. The LPS-induced NF-κB transcriptional activity was elevated many fold in the transfected cells. The treatment of the cells with CA, AA and UA resulted in a significant (P < 0.01) suppression of the LPS-induced luciferase activity when compared with LPS alone (Fig 7).

### CA, AA and UA inhibited IFN-γ secretion from the LPS stimulated macrophages

**Fig 8:**
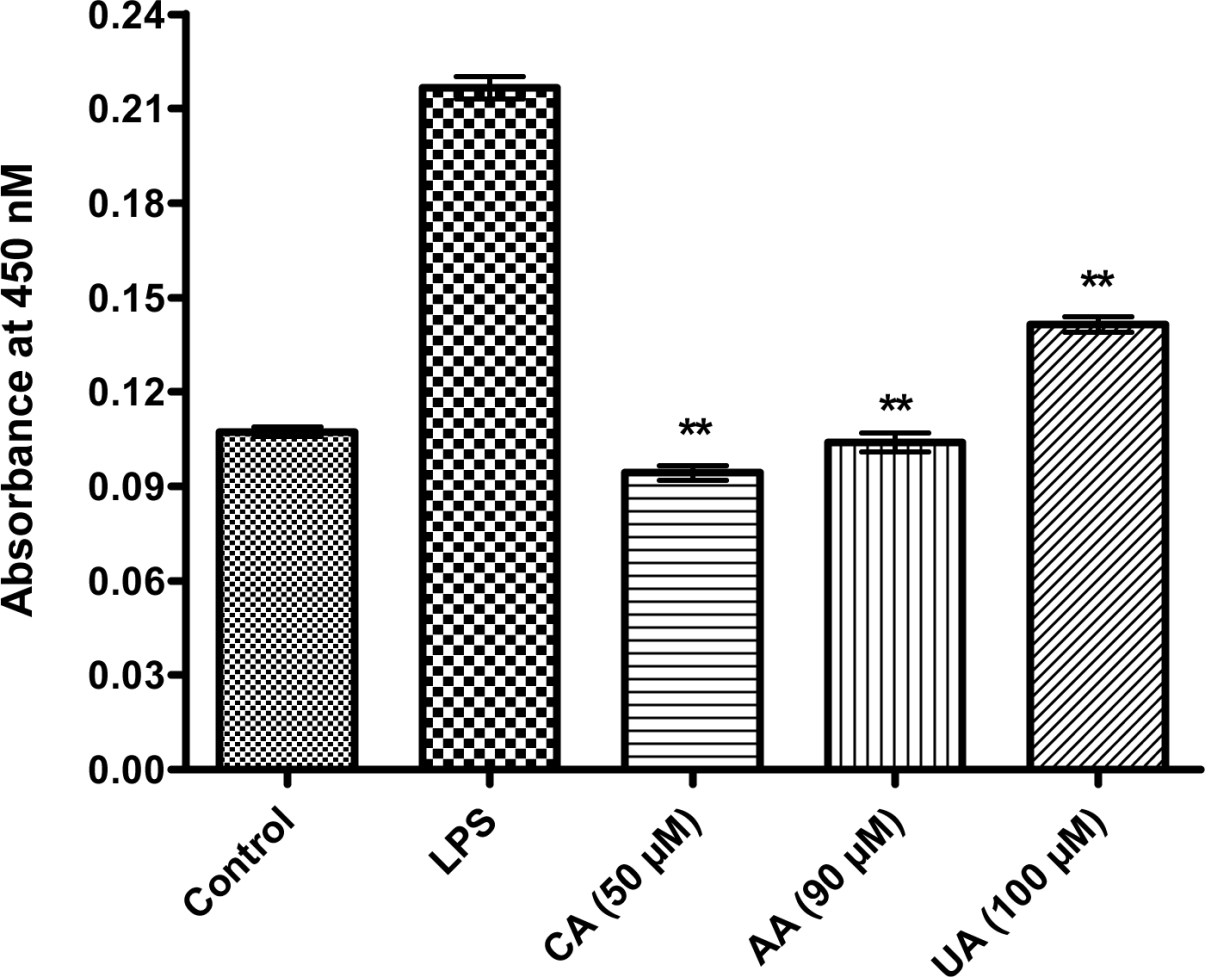
CA, AA and UA inhibited IFN-γ release from LPS stimulated RAW cells. *In vitro* (supernatant) level of IFN-γ in LPS stimulated RAW cells treated with CA, AA and UA detected through indirect ELISA. Y axis represents the absorbance at 450 nm. Data represented as mean ± SD of three independent experiments. **P < 0.01 (ANOVA) as compared with LPS treated group.

The effect of CA, AA and UA on the release of the pro-inflammatory cytokine; IFN-γ from the LPS stimulated macrophages were evaluated by the indirect ELISA method. The expression of the soluble IFN-γ decreased to approximately half in the CA, UA and AA treated cells when compared with the positive controls (Fig 8). The results indicate that the CA, AA and UA could negatively regulate the expression of IFN-γ.

## Discussion

In this study, the screening of a library of 109 PTs through PASS resulted in the identification of 80 molecules with the potential to prevent NF-κB. Further docking study yielded 56 ligands with high docking scores, of which 45 ligands passed the next filter of the softened Lipinski’s Rule of Five. In the cell free hotspot kinase assay, the CA, AA and UA inhibited human IKKβ. As revealed by the results of the western blot analysis, the PTs tested significantly inhibited the IKK-dependent activation of NF-κB in a cellular assay based on the stimulation of the RAW 264.7 cells by the LPS. In the luciferase reporter assay, the CA was found to decrease the luciferase activity most significantly (P < 0.01) at the concentration of 50 μM when compared with the AA (90 μM) and UA (100 μM). The abrogation of NF-κB by these PTs, decreased the expression of the cytokine; IFN-γ. The findings validated the virtual screening approach comprising PASS and the docking simulations of the natural products, mainly the PTs. Moreover, the *in silico* results agree with the *in vitro* results and reported effects of the PTs.

The NF-κB pathway has a central role in the regulation of many physiological, immune-inflammatory and carcinogenic responses. Although, normal activation of NF-κB is desirable, aberrant NF-κB activity is associated with development of pathological conditions [74]. The deregulated NF-κB activity leads to inflammatory bowel disease, Crohn’s disease, neurodegenerative disorders, including Alzheimer’s disease and various types of cancers [25,75]. As the NF-κB is extensively associated with various human diseases, it has become the appropriate therapeutic target of many molecules. Indeed, the search of molecules that inhibit the activity of NF-κB has become an important therapeutic quest [25]. The natural triterpenoids provide an interesting scaffold for new drug development. The NF-κB is one of the important molecular targets of PTs in inflammation and cancer [38,43]. The anticancer activities of PTs appear to be mediated by their common ability to block NF-κB activation [38]. The PTs including CA, AA, UA, boswellic acids, oleanolic acid and many others were the subject of several extensive investigations [76–79]. It has resulted in the establishment of PTs as a promising therapeutic agents for the treatment of various diseases. The ability of CA, AA and UA to inhibit the IKKβ mediated NF-κB activation signifies the potential of these PTs as promising anti-inflammatory and anticancer drugs.

Several studies have reported the NF-κB inhibitory activity of different triterpenoids in the LPS stimulated RAW 264.7 cells [38,40,41]. However, reports on the application of the virtual screening protocols in predicting the effect of the triterpenoids on the NF-κB signaling and their validation through the high throughput and *in-vitro* assays are very limited and need to be explored. The present study was aimed to determine whether the NF-κB modulatory effect of the PTs involves their IKKβ inhibitory activity and intends to confirm the utility of virtual screening in exploring the IKKβ inhibitory PTs through actual biological testing. In present study, a computer program PASS was used to evaluate the general biological potential of a molecule and its predicted biological activities that reflect the compound’s interaction with a biological object based on its structural formula [67,68]. The first step in the virtual screening using PASS indicated that the majority of the PTs exhibit NF-κB inhibitory activity. As the PASS does not predict the direct IKKβ inhibition, Further, we performed the *in silico* docking of the prospective NF-κB inhibitory PTs on the NEMO/IKKβ crystal structure using the prior data on the binding pocket for Withaferin A, considering the structural similarity between the steroidal lactones and the PTs [38,80].

The Glide score XP is an advanced function that penalizes the poses which violate the principles of the established physical chemistry, such as the charged and strongly polar groups that either make an appropriate complement of the hydrogen bonds or are suitably exposed to the solvent [81]. Therefore, docking studies on a limited number of ligands were performed in the more accurate XP docking runs, showing the best XP glide score values (Table 2). The ligands were arranged in the descending order of their glide scores. From the possible docking hits, three molecules, CA, AA and UA were chosen based on their ability to interact selectively with the NEMO/IKKβ complex and the availability of pure compounds from a reliable source. The IKK peptide formed intermolecular hydrogen-bond interactions (SER 85 and GLU 89) with the NEMO while the IKK side chains inside the NEMO pocket formed consolidated intermolecular hydrophobic interactions, which were responsible for the formation of the NEMO/IKKβ complex [10]. The molecules tested fit in the same pocket and interacted with the SER 85 and GLU 89 amino acids. It specifies that the CA, AA and UA have the ability to block the formation of the NEMO/IKKβ complex binding with the same pocket responsible for complex association.

The pharmacokinetic property optimization is a complex task likely to require changes in those molecular determinants that are responsible for the binding affinity and specificity like the hydrogen bonds. The Lipinski’s Rule of Five is a rule of thumb to evaluate drug likeness or to determine if a chemical compound with a certain pharmacological or biological activity has properties that would make it a likely orally active drug for human use. The hydrogen bond acceptor and hydrogen bond donor groups in the compound optimize the drug receptor interactions. The rule describes the delicate balance among the molecular properties of a compound that directly influences its pharmacodynamics, pharmacokinetics and ultimately affects their fate in the human body like a drug [55]. While screening the phytochemicals, the Lipinski’s rule should be unstiffened to avoid over filtering of the compounds, so that the prospective drug candidates are not excluded at the initial stage of drug discovery [49,56,57]. Therefore, in the present study, we applied the softened Lipinski’s Rule of Five. The pharmacokinetic parameters of the PTs selected were within acceptable ranges and demonstrated their likelihood of acting like a drug. All the three compounds fall within the specifications of the softened Rule of Five. Although, glycosides like madecassoside, asiaticoside and glycyrrhizin have top docking scores, they even do not follow the softened Lipinski’s Rule of Five. Hence, they were excluded from further *in vitro* study. The results of the *in silico* study prompted us to screen the selected PTs by the cell free hotspot kinase assay against the human IKKβ. It was observed that all the tested compounds were able to inhibit the human IKKβ, and UA was better in this respect. Although the inhibitory effects of the CA, AA and UA against the human IKKβ in this cell free assay was less prominent when compared with staurosporine, it has no structural resemblance with the triterpenoids and is only used as a reference compound. In our *in-vitro* studies, we tested three compounds, viz., CA, AA and UA, which were included in the list of triterpenoids arranged in the descending order of the docking scores so that the correlation between the docking score against the IKKβ and the actual biological activity could be established. This selection was partially based on the availability of pure compounds from a reliable source. The initial experiments were conducted to establish suitable concentrations of CA, AA and UA that were non-toxic to the RAW cells and used subsequently.

Macrophages are responsible for the release of various inflammatory mediators and play a central role in human immune defence mechanisms [82–84]. The prototypical endotoxin LPS stimulates the Toll-like receptor 4 (TLR4) located on the macrophage surfaces and produces inflammatory molecules [85,86]. It triggers the subsequent activations of the downstream signaling pathways, of which the NF-κB signaling is the main pathway. LPS activates the IκB phosphorylation and degradation process through IκB kinase (IKK), predominantly the IKKβ, which facilitates the release and nuclear translocation of the NF-κB which leads to an increased transcription of the pro-inflammatory cytokines and inflammation [71,72,87]. The reduction of the LPS-inducible inflammatory mediators is one of the attractive therapeutic strategies for many acute and chronic inflammatory diseases [40].

We observed the inhibition of the NF-κB and IKKα/β phosphorylation by the CA was approximately one-fold and two-fold, respectively. It implies that suppression of the NF-κB by CA is mediated through the inhibition of the IKKβ phosphorylation, which is responsible for the activation of the NF-κB. Earlier studies on the CA in apolipoprotein E-deficient mice reported the inhibition of NF-κB along with the down-regulation of the other genes and our results are consistent with the previous observation [88]. The results of Western blot support that the inhibition of the NF-κB by CA is mainly due to the inhibition of IKKβ phosphorylation. Checker et al. [79] also found inhibition of NF-κB expression and NF-κB dependent gene products in activated lymphocytes by ursolic acid. After LPS stimulation, the NF-κB becomes activated and translocate from the cytoplasm to the nucleus. The translocation of the NF-κB to the nucleus was inhibited by CA, AA and UA as evident from the reduced expression of the NF-κB in the nuclear fractions of LPS stimulated RAW 264.7 cells, after pretreatment with the PTs (Fig 7).

**Fig 9:**
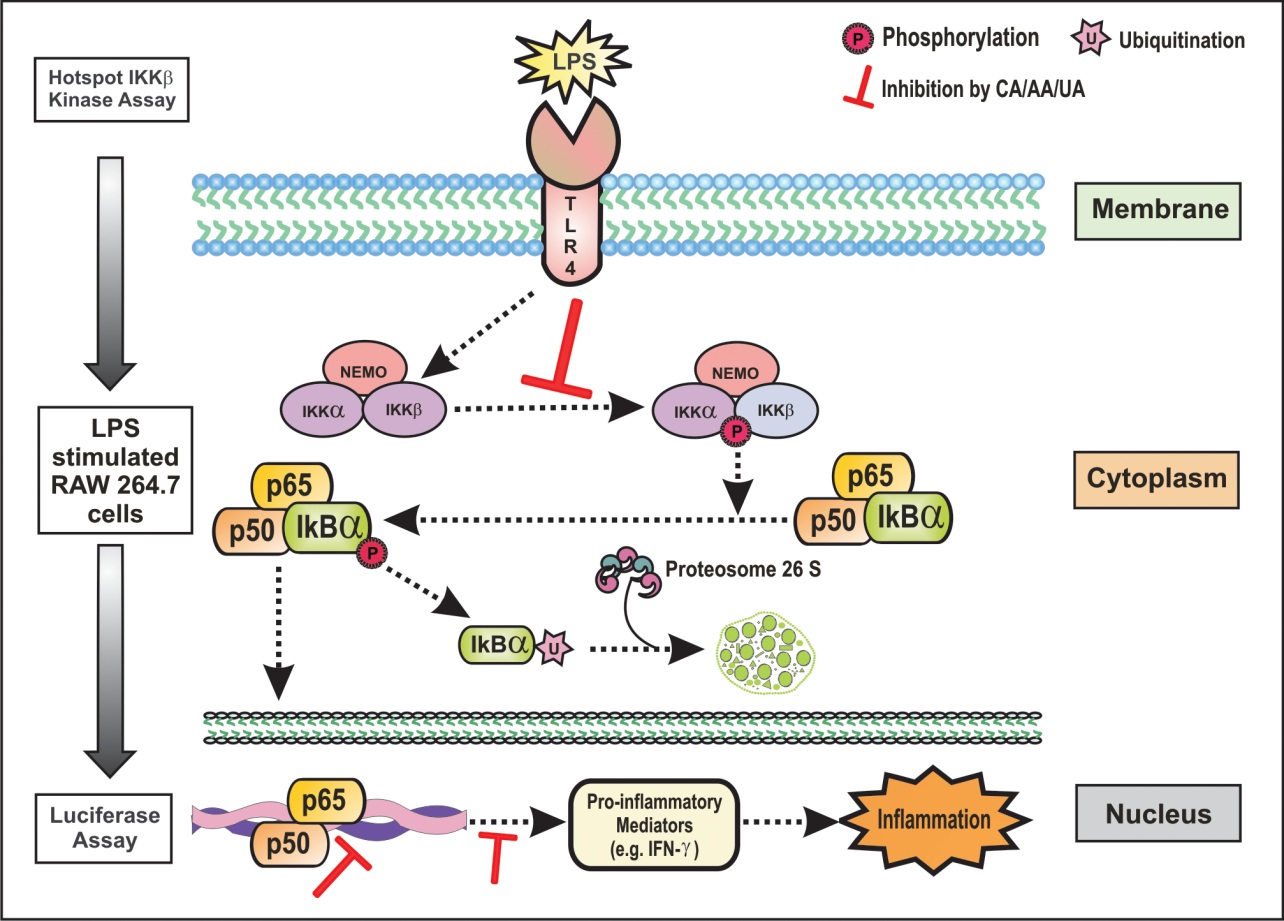
Proposed mechanism of action of pentacyclic triterpenoids on LPS induced NF-κB signaling. Abrogation of NF-κB activation by pentacyclic triterpenoids may be resulted due to down regulated IKKβ phosphorylation.

As the IKKs (especially the IKKβ) are extensively involved in the inflammatory stimuli-mediated NF-κB activation, it is targetable through the pharmacological intervention in numerous inflammatory diseases [89]. It is noted that the CA, AA and UA inhibited the expression of the phosphorylated IKKβ, which suggests that the inhibition of NF-κB activation by the PTs tested here in this study results from an interrupted IKK activation. Reporter gene analyses further clearly revealed that the CA, AA and UA selectively inhibited the NF-κB activation occurred due to the LPS treatment. Among the PTs tested in the cellular assay by western blot in LPS stimulated RAW 264.7 macrophages, CA appears to decrease the nuclear expressions of the NF-κB and phospho-IKKβ more potently at concentrations ranging from 20 to 70 μM. The AA and UA also demonstrated effects similar to CA although at somewhat higher (70–120 μM) concentrations. The increase in the IKK/NF-κB activity has a critical role in LPS stimulated inflammation. The pretreatment of the LPS treated mice with the IKK inhibitor XII decreases the plasma levels of the IFN-γ, a pro-inflammatory cytokine [90]. This study substantiates the significance of the IKK inhibition in delaying the proinflammatory response in the LPS-treated mice. We determined the IFN-γ levels in the culture supernatants of the LPS-stimulated mouse macrophages. The CA, AA and UA significantly (P < 0.01) decreased the expression of the pro-inflammatory cytokine, IFN-γ. This is an additional evidence to indicate the ability of the PTs to inhibit the IKK-mediated NF-κB activation and the subsequent suppression of the pro-inflammatory cytokines. Our findings are in accordance with the results from an earlier *in vivo* study in the LPS-treated mice [90]. Modulation of the LPS-induced NF-κB signaling by the PTs along with the proposed mechanisms is presented as schematics in Fig 9.

The present study revealed that the CA, AA and UA inhibit the LPS-induced expression of the NF-κB, IKKα, Phospho-IKKα/β and Akt in RAW 264.7 cells. The NF-κB which controls the several genes involved in inflammation and immunity is activated by the LPS [4]. The inhibition of the NF-κB through the blockade of the IKKβ can reduce the expression of the proinflammatory genes. The NF-κB activation is regulated by several cellular kinases such as Mitogen Activated Protein Kinases (MAPKs), which include p38, the extracellular signal responsive kinase (ERK1/2) and the c-Jun N-terminal kinase (JNK) subgroups [41,87,91]. Moreover, it is important to establish the relative contribution of these kinases to LPS-induced signaling in the macrophages. However, the aim of this study was to check the effect of the PTs on IKKβ mediated inhibition of the NF-κB activation. We examined the expression of selected proteins, in relation to the NF-κB cascade. Taken together, the findings of the *in silico* and protein expression studies showed that treatment with CA, AA and UA suppress the NF-κB activation initially via the IKKβ-dependent mechanism. However, the triterpenoids also interact with other intermediates in the NF-κB signaling pathway, like caspases, Bcl-2, PARP, STAT, Bax, PKC, MEPK, ERK etc. [38]. The role of such intermediates in the observed NF-κB inhibitory activity cannot be negated at this stage.

In summary, an approach involving the sequential filtering of compounds through PASS, docking study and *in vitro* assays was employed in the present study. The CA, AA and UA selected through the initial filters of PASS, molecular docking and softened Lipinski’s Rule of Five were found to inhibit the NF-κB activity. The current study demonstrates that the NF-κB inhibitor PTs exert their action through the inhibition of the IKKβ kinase activity and its phosphorylation. This class of molecules can be further explored as potent IKKβ inhibitors. Although, the pharmacological and pharmacokinetic predictions of the PTs tested support their drug likeliness, further studies elaborating the pharmacokinetics of these potential compounds are required. Among these three extensively tested PTs, CA was the most potent inhibitor of the NF-κB signaling. The chemical alterations of the triterpenoids, considering their binding site on the NEMO-IKKβ binding domain and the recently reported IKKβ crystal structure, can accelerate the discovery of new NF-κB signaling inhibitors. The *in silico* results of the current study can assist in drawing up the design of the virtual screening protocols for the natural products that inhibit the IKKβ mediated NF-κB signaling pathway and offer insights into the discovery of new kinase inhibitors. The PTs including CA, UA and AA may be used as leads in developing drugs for the treatment of diseases involving immune-inflammatory perturbations. Correlation studies involving the pharmacokinetics and pharmacodynamics on the synthetic or semi-synthetic derivatives of these PTs may yield compounds with better therapeutic potential.

## Supporting Information

S1 Fig High throughput virtual screening workflow. (TIF)

S1 Table Compound library of pentacyclic triterpenoids. (DOC)

S2 Table Softened Lipinski’s rule of five for drug likeliness and *in silico* ADME properties of PTs by QikProp (Schordinger 9.0). (DOC)

## References

[1] Bonizzi G, Karin M. The two NF-κB activation pathways and their role in innate and adaptive immunity. Trends Immunol. 2004; 25(6): 280–288. (10.1016/j.it.2004.03.008) 15145317

[2] Ghosh S, May MJ, Kopp EB. NF-κB and Rel proteins: evolutionary conserved mediators of immune responses. Annu Rev Immunol. 1998; 16: 225–260. (10.1146/annurev.immunol.16.1.225) 9597130

[3] Li Q, Verma IM. NF-kappaB regulation in the immune system. Nat Rev Immunol. 2002; 2(10): 725–734. (10.1038/nri910) 12360211

[4] Hoesel B, Schmid JA. The complexity of NF-κB signaling in inflammation and cancer. Mol Cancer. 2013; 12: 86 (10.1186/1476-4598-12-86) 23915189 PMC3750319

[5] Sun SC, Chang JH, Jin J. Regulation of nuclear factor-κB in autoimmunity. Trends Immunol. 2013; 34(6): 282–289. (10.1016/j.it.2013.01.004) 23434408 PMC3664242

[6] Gilmore TD. Introduction to NF-κB: players, pathways, perspectives. Oncogene. 2006; 25(51): 6680–6684. (10.1038/sj.onc.1209954) 17072321

[7] Karin M. How NF-kappaB is activated: the role of the IκB kinase (IKK) complex. Oncogene.1999; 18(49): 6867–6874. (10.1038/sj.onc.1203219) 10602462

[8] Scheidereit C. IkappaB kinase complexes: gateways to NF-kappaB activation and transcription. Oncogene. 2006; 25(51): 6685–6705. (10.1038/sj.onc.1209934) 17072322

[9] Tergaonkar V. NF-kappaB pathway: a good signaling paradigm and therapeutic target. Int J Biochem Cell Biol. 2006; 38(10): 1647–1653. (10.1016/j.biocel.2006.03.023) 16766221

[10] Grover A, Shandilya A, Punetha A, Bisaria VS, Sundar D. Inhibition of the NEMO/IKKβ association complex formation, a novel mechanism associated with the NF-κB activation suppression by *Withania somnifera’s* key metabolite withaferin A. BMC Genomics. 2010; 11(Suppl 4): S25 (10.1186/1471-2164-11-S4-S25) 21143809 PMC3005936

[11] Lee SH, Toth Z, Wong LY, Brulois K, Nguyen J, Lee JY, et al Novel phosphorylations of IKKγ/NEMO. MBio. 2012; 3(6): e00411–12. (10.1128/mBio.00411-12) 23131831 PMC3487776

[12] Leung CH, Chan DS, Li YW, Fong WF, Ma DL. Hit identification of IKKβ natural product inhibitor. BMC Pharmacol Toxicol. 2013; 14:3 (10.1186/2050-6511-14-3) 23294515 PMC3583241

[13] Tak PP, Firestein GS. NF-kappaB: a key role in inflammatory diseases. J Clin Invest. 2001; 107(1): 7–11. (10.1172/JCI11830) 11134171 PMC198552

[14] Cha DS, Eun JS, Jeon H. Anti-inflammatory and antinociceptive properties of the leaves of *Eriobotrya japonica* . J Ethnopharmacol. 2011; 134(2): 305–312. (10.1016/j.jep.2010.12.017) 21182921

[15] Rothgiesser KM, Erener S, Waibel S, Luscher B, Hottiger MO. SIRT2 regulates NF-κB-dependent gene expression through deacetylation of P65 Lys310. J Cell Sci. 2010; 123(24): 4251–4258. (10.1242/jcs.073783) 21081649

[16] Bamborough P, Cllahan JF, Christopher JA, Kerns JK, Liddle JL, Miller DD, et al Progress towards the development of anti-inflammatory inhibitors of IKKβ. Curr Top Med Chem. 2009; 9(7): 623–639. (10.2174/156802609789007336) 19689370

[17] Gupta SC, Sundaram C, Reuter S, Aggarwal BB. Inhibiting NF-κB activation by small molecules as a therapeutic strategy. Biochim Biophys Acta. 2010; 1799(10–12): 775–787. (10.1016/j.bbagrm.2010.05.004) 20493977 PMC2955987

[18] Kim JH, Lee G, Cho YL, Kim CK, Han S, Lee H, et al Desmethylanhydroicaritin inhibits NF-κB-regulated inflammatory gene expression by modulating the redox-sensitive PI3K/PTEN/Akt pathway. Eur J Pharmacol. 2009; 602(2–3): 422–431. (10.1016/j.ejphar.2008.10.062) 19027002

[19] Mbalaviele G, Sommers CD, Bonar SL, Mathialagan S, Schindler JF, Guzova JA, et al Novel, highly selective, tight binding IκB kinase-2 (IKK-2) inhibitor: a tool to correlate IKK-2 activity to the fate and functions of the components of the nuclear factor-κB pathway in arthritis-relevant cells and animal models. J Pharmacol Exp Ther. 2009; 329(1): 14–25. (10.1124/jpet.108.143800) 19168710

[20] Wen D, Nong Y, Morgan JG, Gangurde P, Bielecki A, Dasilva J, et al A selective small molecule IκB kinase β inhibitor blocks nuclear factor κB-mediated inflammatory responses in human fibroblast-like synoviocytes, chondrocytes, and mast cells. J Pharmacol Exp Ther. 2006; 317(3): 989–1001. (10.1124/jpet.105.097584) 16525037

[21] Nagarajan S, Choo H, Cho YS, Shin KJ, Oh KS, Lee BH, et al IKKβ inhibitor identification: a multi-filter driven novel scaffold. BMC Bioinformatics. 2010; 11(Suppl 7):S15 (http://dx.doi.org/S15. 10.1186/1471-2105-11-S7-S15) 21106122 PMC2957683

[22] Nam KY, Oh WS, Kim C, Song MY, Jougn JY, Kim SY, et al Computational drug discovery approach based on nuclear factor-κB pathway dynamics. Bull Korean Chem Soc. 2011; 32(12): 4397–4402. (10.5012/bkcs.2011.32.12.4397)

[23] Xu G, Lo YC, Li Q, Napolitano G, Wu X, Jiang X, et al Crystal structure of inhibitor of κB kinase β. Nature. 2011; 472: 325–330. (10.1038/nature09853) 21423167 PMC3081413

[24] Huang JJ, Wu XW, Jia JM, Guo XK, Xue X, Jiang ZY, et al Novel IKK inhibitors discovery based on the co-crystal structure by using binding-conformation-based and ligand-based method. Eur J Med Chem. 2013; 63: 269–278. (10.1016/j.ejmech.2013.01.045) 23501112

[25] Heras BDL, Hortelano S. Molecular basis of the anti-inflammatory effects of terpenoids. Inflamm Allergy Drug Targets. 2009; 8(1): 28–39. (10.2174/187152809787582534) 19275691

[26] Tripathi P. Aggarwal A. NF-κB transcription factor: a key player in the generation of immune response. Current Science. 2006; 90(4): 519–531.

[27] Yamamoto Y, Gaynor RB. Therapeutic potential of inhibition of the NF-kappaB pathway in the treatment of inflammation and cancer. J Clin Invest. 2001; 107(2): 135–142. (10.1172/JCI11914) 11160126 PMC199180

[28] Verma I. Nuclear factor (NF)-κB proteins: therapeutic targets. Ann Rheum Dis. 2004; 63(Suppl 2): ii57–ii61. (10.1136/ard.2004.028266) 15479873 PMC1766777

[29] Dzubak P, Hajduch M, Vydra D, Hustova A, Kvasnica M, Biedermann D, et al Pharmacological activities of natural triterpenoids and their therapeutic implications. Nat Prod Rep. 2006; 23(3): 394–411. (doi: http://dx.doi.org/10.1039/B515312N) 16741586 10.1039/b515312n

[30] Alqahtani A, Hamid K, Kam A, Wong KH, Abdelhak Z, Razmovski-Naumovski V, et al The pentacyclic triterpenoids in herbal medicines and their pharmacological activities in diabetes and diabetic complications. Curr Med Chem. 2013; 20(7): 908–931. (10.2174/0929867311320070007) 23210780

[31] Jager S, Trojan H, Kopp T, Laszczyk MN, Scheffler A. Pentacyclic triterpene distribution in various plants—rich sources for a new group of multi-potent plant extracts. Molecules. 2009; 14(6): 2016–2031. (10.3390/molecules14062016) 19513002 PMC6254168

[32] Laszczyk MN. Pentacyclic triterpenes of the lupane, oleanane and ursane group as tools in cancer therapy. Planta Med. 2009; 75(15): 1549–1560. (10.1055/s-0029-1186102) 19742422

[33] Safayhi H, Sailer ER. Anti-inflammatory actions of pentacyclic triterpenes. Planta Med. 1997; 63(6): 487–493. (10.1055/s-2006-957748) 9434597

[34] Calixo JB, Otuki MF, Santos ARS. Anti-inflammatory compounds of plant origin. Part I. Action on Arachidonic Acid Pathway, Nitric Oxide and Nuclear Factor κB (NF-κB). Planta Med. 2003; 69(11): 973–983. (10.1055/s-2003-45141) 14735432

[35] Gautam R, Jachak SM. Recent developments in anti-inflammatory natural products. Med Res Rev. 2009; 29(5): 767–820. (10.1002/med.20156) 19378317

[36] Rios JL. Effects of triterpenes on the immune system. J Ethnopharmacol. 2010; 128(1): 1–14. (10.1016/j.jep.2009.12.045) 20079412

[37] Salminen A, Lehtonen M, Suuronen T, Kaarniranta K, Huuskonen J. Terpenoids: natural inhibitors of NF-kappaB signalling with anti-inflammatory and anticancer potential. Cell Mol Life Sci. 2008; 65(19): 2979–2999. (10.1007/s00018-008-8103-5) 18516495 PMC11131807

[38] Yadav VR, Prasad S, Sung B, Kannappan R, Aggarwal BB. Targeting inflammatory pathways by triterpenoids for prevention and treatment of cancer. Toxins. 2010; 2(10): 2428–2466. (10.3390/toxins2102428) 22069560 PMC3153165

[39] Yore MM, Liby KT, Honda T, Gribble GW, Sporn MB. The synthetic triterpenoid 1-[2-cyano-3,12-dioxooleana-1,9(11)-dien-28-oyl]imidazole blocks nuclear factor-κB activation through direct inhibition of IκB kinase B. Mol Cancer Ther. 2006; 5: 3232–3239. (10.1158/1535-7163.MCT-06-0444) 17148759

[40] Kim IT, Ryu S, Shin JS, Choi JH, Park HJ, Lee KT. Euscaphic acid isolated from roots of *rosa rugosa* inhibits LPS-induced inflammatory responses via TLR4-mediated NF-κB inactivation in RAW 264.7 macrophages. J Cell Biochem. 2012; 113(6): 1936–1946. (10.1002/jcb.24062) 22234926

[41] Lee JH, Koo TH, Yoon H, Jung HS, Jin HZ, Lee K, et al Inhibition of NF-κB Activation through targeting I kappa B kinase by celastrol, a quinone methide triterpenoid. Biochemical Pharmacol. 2006; 72(10): 1311–1321. (10.1016/j.bcp.2006.08.014) 16984800

[42] Lu J, Wu DM, Zheng YL, Hu B, Cheng W, Zhang ZF, et al Ursolic acid improves high fat diet-induced cognitive impairments by blocking endoplasmic reticulum stress and IκB kinase B/nuclear factor-κB-mediated inflammatory pathways in mice. Brain Behav Immun. 2011; 25(8): 1658–1667. (10.1016/j.bbi.2011.06.009) 21708244

[43] Sporn MB, Liby K, Yore MM, Suh N, Albini A, Honda T, et al Platforms and networks in triterpenoid pharmacology. Drug Dev Res. 2007; 68(4): 174–182. (10.1002/ddr.20179)

[44] Salminen A, Lehtonen M, Paimela T, Kaarnirantaet K. Celastrol: molecular targets of thunder god vine. Biochem Biophys Res Commun. 2010; 394(3): 439–442. (10.1016/j.bbrc.2010.03.050) 20226165

[45] Goel RK, Singh D, Lagunin A, Poroikov V. PASS-assisted exploration of new therapeutic potential of natural products. Med Chem Res. 2011; 20(9): 1509–1514. (10.1007/s00044-010-9398-y)

[46] Filimonov DA, Lagunin AA, Gloriozova TA, Rudik AV, Druzhilovskii DS, Pogodin PV, et al Prediction of the biological activity spectra of organic compounds using the PASS online web resource. Chemistry of Heterocyclic Compounds. 2014; 50(3): 444–457. (10.1007/s10593-014-1496-1)

[47] Lagunin AA, Goel RK, Gawande DY, Pahwa P, Gloriozova TA, Dmitriev AV, et al Chemo and bioinformatics resources for in silico drug discovery from medicinal plants beyond their traditional use: a critical review. Nat Prod Rep. 2014; 31: 1585–1611. (10.1039/C4NP00068D) 25051191

[48] Chen H, Yao K, Nadas J, Bode AM, Malakhova M, Oi N, et al Prediction of molecular targets of cancer preventing flavonoid compounds using computational methods. PLoS One. 2012; 7(5): e38261 (10.1371/journal.pone.0038261) 22693608 PMC3365021

[49] Petit J, Meurice N, Kaiser C, Maggiora G. Softening the rule of five-where to draw the line? Bioorg Med Chem. 2012; 20(18): 5343–5351. (10.1016/j.bmc.2011.11.064) 22222160 PMC4209914

[50] LigPrep, version 2.3, Schrodinger, LLC, New York, 2009.

[51] Lagunin A, Filimonov D, Poroikov V. Multi-targeted natural products evaluation based on biological activity prediction with PASS. Curr Pharm Des. 2010; 16(15): 1703–1717. (10.2174/138161210791164063) 20222853

[52] Glide, version 5.5, Schrodinger, LLC, New York, 2009.

[53] QikProp, Version 9.0, Schrodinger, LLC, New York, 2009.

[54] Halgren TA. Merck molecular force field. II. MMFF94 van der Waals and electrostatic parameters for intermolecular interactions. J Comput Chem. 2010; 17(5–6): 520–552. (10.1002/(SICI)1096-987X(199604)17:5/6<520::AID-JCC2>3.0.CO;2-W)

[55] Lipinski CA, Lombardo F, Dominy BW, Feeney PJ. Experimental and computational approaches to estimate solubility and permeability in drug discovery and development settings. Adv Drug Deliv Rev. 2001; 46(1–3): 3–26. (10.1016/S0169-409X(00)00129-0) 11259830

[56] Tolliday N, Clemons PA, Ferraiolo P, Koehler AN, Lewis TA, Li X, et al Small molecules, big players: the national cancer institute’s initiative for chemical genetics. Cancer Res. 2006; 66(18): 8935–8942. (10.1158/0008-5472.CAN-06-2552) 16982730

[57] Zhang MQ, Wilkinson B. Drug discovery beyond the ‘rule-of-five’. Curr Opin Biotechnol. 2007; 18(6): 478–488. (10.1016/j.copbio.2007.10.005) 18035532

[58] Anastassiadis T, Deacon SW, Devarajan K, Ma H, Peterson JR. Comprehensive assay of kinase catalytic activity reveals features of kinase inhibitor selectivity. Nat Biotechnol. 2011; 29 (11): 1039–1045. (10.1038/nbt.2017) 22037377 PMC3230241

[59] Knight ZA, Shokat KM. Features of selective kinase inhibitors. Chem Biol. 2005; 12(6): 621–637. (10.1016/j.chembiol.2005.04.011) 15975507

[60] Chakraborty P, Saraswat G, Kabir SN. α-Dihydroxychalcone-glycoside (α-DHC) isolated from the heartwood of *pterocarpus marsupium* inhibits LPS induced MAPK activation and upregulates HO-1 expression in murine RAW 264.7 macrophage. Toxicol Appl Pharmacol. 2014; 277(1): 95–107. (10.1016/j.taap.2014.03.011) 24675710

[61] Cao W, Li XQ, Zhang XN, Hou Y, Zeng AG, Xie YH, et al Madecassoside suppresses LPS-induced TNF-α production in cardiomyocytes through inhibition of ERK, P38, and NF-κB activity. Int Immunopharmacol. 2010; 10(7): 723–729. (10.1016/j.intimp.2010.03.015) 20381648

[62] Preet R, Mohapatra P, Mohanty S, Sahu SK, Choudhuri T, Wyatt MD, et al Quinacrine has anticancer activity in breast cancer cells through inhibition of topoisomerase activity. Int J Cancer. 2012; 130(7): 1660–1670. (10.1002/ijc.26158) 21544805

[63] An HJ, Kim IT, Park HJ, Kim HM, Choi JH, Lee KT. Tormentic acid, a triterpenoid saponin, isolated from *Rosa rugosa*, inhibited LPS-induced iNOS, COX-2, and TNF-α expression through inactivation of the nuclear factor-κB pathway in RAW 264.7 macrophages. Int Immunopharmacol. 2011; 11(4): 504–510. (10.1016/j.intimp.2011.01.002) 21237302

[64] Satapathy SR, Mohapatra P, Preet R, Das D, Sarkar B, Choudhuri T, et al Silver-based nanoparticles induce apoptosis in human colon cancer cells mediated through p53. Nanomedicine. 2013; 8(8): 1307–1322. (10.2217/nnm.12.176) 23514434

[65] Moola ZB, Scawen MD, Atkinson T, Nicholls DJ. Erwinia chrysanthemi L-asparaginase: epitope mapping and production of antigenically modified enzymes. Biochem J. 1994; 302: 921–927. 7945221 10.1042/bj3020921 PMC1137318

[66] Filz O, Lagunin A, Filimonov D, Poroikov V. Computer-aided prediction of QT-prolongation. SAR QSAR Environ Res. 2008; 19(1–2): 81–90. (10.1080/10629360701844183) 18311636

[67] Lagunin A, Filimonov D, Zakharov A, Xie W, Huang Y, Zhu F, et al Computer-aided prediction of rodent carcinogenicity by PASS and CISOC-PSCT. QSAR Comb Sci. 2009; 28(8): 806–810. (10.1002/qsar.200860192)

[68] Parasuraman S. Prediction of biological spectra of substances. J Pharmacol Pharmacother. 2011; 2(1): 52–53. (10.4103/0976-500X.77119) 21701651 PMC3117574

[69] Kryzhanovskii SA, Salimov RM, Lagunin AA, Filimonov DA, Gloriozova TA, Poroikov VV. Nootropic action of some antihypertensive drugs: computer predicting and experimental testing. Pharm Chem J. 2012; 45(10): 605–611. (10.1007/s11094-012-0689-0)

[70] Wisdom R, Johnson RS, Moore C. C-Jun regulates cell cycle progression and apoptosis by distinct mechanisms. EMBO J. 1999; 18(1): 188–197. (10.1093/emboj/18.1.188) 9878062 PMC1171114

[71] Choi YH, Jin GY, Li GZ, Yan GH. Cornuside suppresses lipopolysaccharide-induced inflammatory mediators by inhibiting nuclear factor-kappa B activation in RAW 264.7 macrophages. Biol Pharm Bull. 2011; 34(7): 959–966. (10.1248/bpb.34.959) 21719998

[72] Fengyang L, Yunhe F, Bo L, Zhicheng L, Depeng L, Dejie L, et al Stevioside suppressed inflammatory cytokine secretion by downregulation of NF-κB and MAPK signaling pathways in LPS-stimulated RAW264.7 cells. Inflammation. 2012; 35(5): 1669–1675. (10.1007/s10753-012-9483-0) 22644339

[73] Jung YS, Kim DH, Hwang JY, Yun NY, Lee YH, Han SB, et al Anti-inflammatory effect of tricin 4′-O-(threo-β-guaiacylglyceryl) ether, a novel flavonolignan compound isolated from Njavara on in RAW264.7 cells and in ear mice edema. Toxicol Appl Pharmacol. 2014; 277: 67–76. (10.1016/j.taap.2014.03.001) 24631338

[74] Aggarwal BB, Takada Y, Shishodia S. Nuclear transcriptional factor NF-kappa B: Role in biology and medicine. Indian J Exp Biol. 2004; 42(4): 341–353. 15088683

[75] Wong ET, Tergaonkar V. Roles of NF-kappaB in health and disease: mechanisms and therapeutic potential. Clin Sci (Lond). 2009; 116(6): 451–465. (10.1042/CS20080502) 19200055

[76] Shah BA, Qazi GN, Taneja SC. Boswellic acids: a group of medicinally important compounds. Nat Prod Rep. 2009; 26(1): 72–89. (10.1039/B809437N) 19374123

[77] Liu J. Pharmacology of oleanolic acid and ursolic acid. J Ethnopharmacol. 1995; 49(2): 57–68. (10.1016/0378-8741(95)90032-2) 8847885

[78] Pollier J, Goossens A. Oleanolic acid. Phytochemistry. 2012; 77: 10–15. (10.1016/j.phytochem.2011.12.022) 22377690

[79] Checker R, Sandur SK, Sharma D, Patwardhan RS, Jayakumar S, Kohli V, et al Potent anti-inflammatory activity of ursolic acid, a triterpenoid antioxidant, is mediated through suppression of NF-κB, AP-1 and NF-AT. PLoS One. 2012; 7(2): e31318 (10.1371/journal.pone.0031318) 22363615 PMC3282718

[80] Chen RJ, Chung TY, Li FY, Yang WH, Jinn TR, Tzen JT. Steroid-like compounds in chinese medicines promote blood circulation via inhibition of Na+/K+-ATPase. Acta Pharmacol Sin. 2010; 31(6): 696–702. (10.1038/aps.2010.61) 20523340 PMC4002980

[81] Piccagli L, Borgatti M, Nicolis E, Bianchi N, Mancini I, Lampronti I, et al Virtual screening against nuclear factor-κB (NF-κB) of a focus library: identification of bioactive furocoumarin derivatives inhibiting NF-κB dependent biological functions involved in cystic fibrosis. Bioorg Med Chem. 2010; 18(23): 8341–8349. 10.1016/j.bmc.2010.09.063) 20980154

[82] Klimpel GR. Immune Defenses In: Baron S, editor. Medical microbiology. 4th edition Galveston (TX): University of Texas Medical Branch at Galveston; 1996. Chapter 50. 21413332

[83] Moura IF, Silva L, Leal EC, Tellechea A, Cruz MT, Carvalho E. Neurotensin modulates the migratory and inflammatory response of macrophages under hyperglycemic conditions. Biomed Res Int. 2013; 2013: 941764 10.1155/2013/941764) 24000330 PMC3755412

[84] West MA, Seatter SC, Bellingham J, Clair L. Mechanisms of reprogrammed macrophage endotoxin signal transduction after lipopolysaccharide pretreatment. Surgery. 1995; 118(2): 220–228. 10.1016/S0039-6060(05)80327-7) 7638737

[85] Kwon OK, Lee MY, Yuk JE, Oh SR, Chin YW, Lee HK, et al Anti-Inflammatory effects of methanol extracts of the root of *Lilium lancifolium* on LPS-stimulated RAW264.7 cells. J Ethnopharmacol. 2010; 130(1): 28–34. 10.1016/j.jep.2010.04.002) 20412846

[86] Nathan C. Nitric oxide as a secretory product of mammalian cells. FASEB J. 1992; 6(12): 3051–3064. 1381691

[87] Choi RJ, Chun J, Khan S, Kim YS. Desoxyrhapontigenin, A potent anti-inflammatory phytochemical, inhibits LPS-induced inflammatory responses via suppressing NF-κB and MAPK pathways in RAW 264.7 Cells. Int Immunopharmacol. 2014; 18(1): 182–190. 10.1016/j.intimp.2013.11.022) 24295651

[88] Chen H, Yang J, Zhang Q, Chen LH, Wang Q. Corosolic acid ameliorates atherosclerosis in apolipoprotein E-deficient mice by regulating the nuclear factor-κB signaling pathway and inhibiting monocyte chemoattractant protein-1 expression. Circ J. 2012; 76(4): 995–1003. 10.1253/circj.CJ-11-0344) 22293444

[89] Karin M, Delhase M. The I kappa B kinase (IKK) and NF-kappa B: key elements of proinflammatory signalling. Semin Immunol. 2000; 12(1): 85–98. 10.1006/smim.2000.0210) 10723801

[90] Novoselova EG, Khrenov MO, Glushkova OV, Lunin SM, Parfenyuk SB, Novoselova TV, et al Anti-inflammatory effects of IKK inhibitor XII, thymulin, and fat-soluble antioxidants in LPS-treated mice. Mediators Inflamm. 2014; 2014: 724838 10.1155/2014/724838) 25045213 PMC4089567

[91] Ruland J, Mak TW. Transducing signals from antigen receptors to nuclear factor kappaB. Immunol Rev. 2003; 193(1): 93–100. 10.1034/j.1600-065X.2003.00049.x) 12752674

