# Supplementary figures and images for: Pentacyclic Triterpenoids Inhibit IKKβ Mediated Activation of NF-κB Pathway: In Silico and In Vitro Evidences
Source: PLoS One. 2015 May 4;10(5):e0125709. doi: 10.1371/journal.pone.0125709 (PMC4418667; doi:10.1371/journal.pone.0125709)

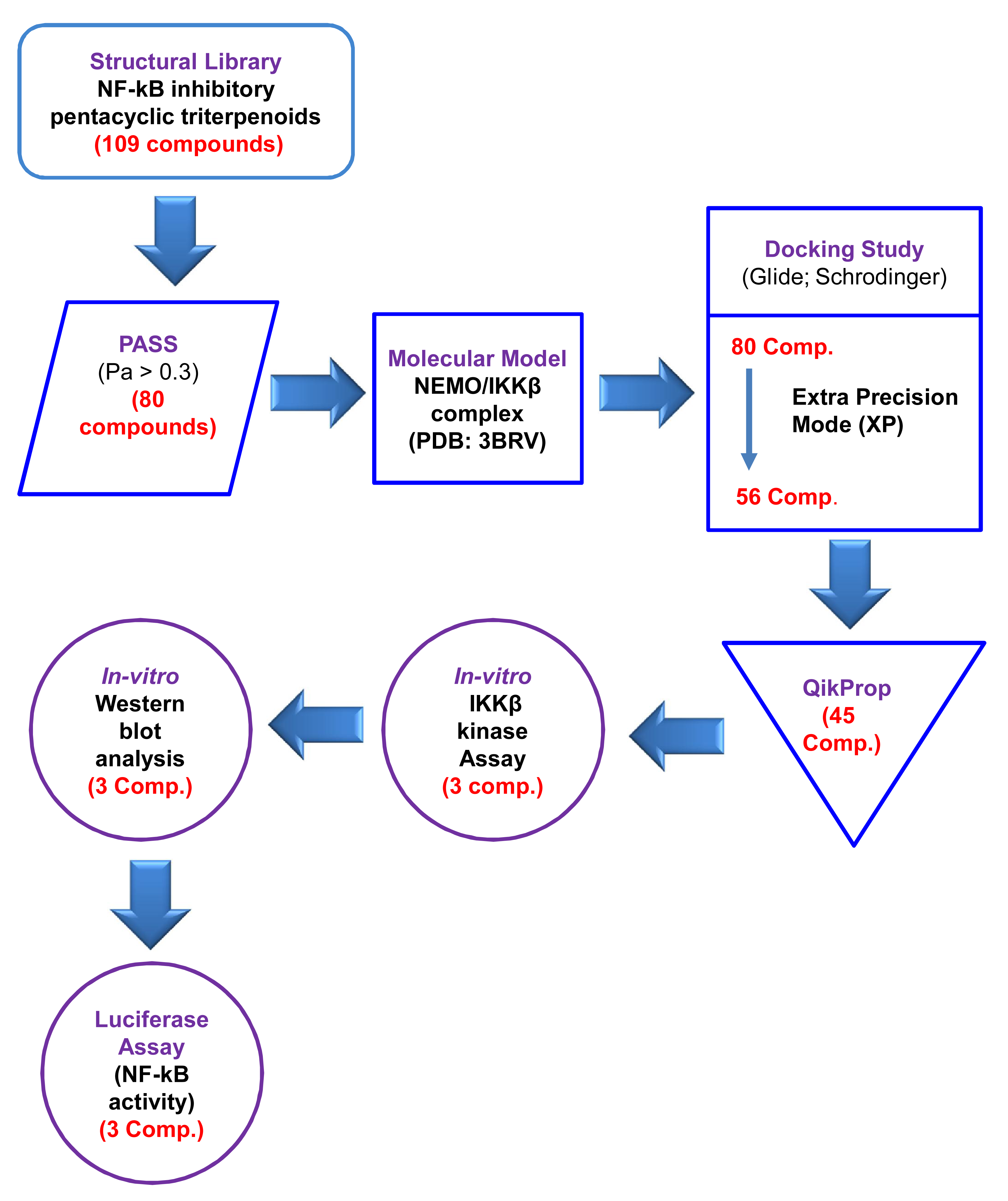

Supplement: S1 Fig — (TIF) [file pone.0125709.s001.tif]
