# Supplementary material for: Pentacyclic Triterpenoids Inhibit IKKβ Mediated Activation of NF-κB Pathway: In Silico and In Vitro Evidences
Source: PLoS One. 2015 May 4;10(5):e0125709. doi: 10.1371/journal.pone.0125709 (PMC4418667; doi:10.1371/journal.pone.0125709)
Supplement: S2 Table — (DOC) [file pone.0125709.s003.doc]

**S2 Table: Softened Lipinski’s rule of ﬁve for drug likeliness and *in silico* ADME properties of PTs by QikProp (Schordinger 9.0).**

| **Sr. No.** | **Compound** | **Softened Lipinski’s rule of ﬁve (Drug Likeliness)** | | | | | **In silico ADME by QikProp** | | | | |
| --- | --- | --- | --- | --- | --- | --- | --- | --- | --- | --- | --- |
|  |  | **Violations of Lipinski’s**  **Rule of five^a^** | **Molecular**  **Weight^b^** | **QPlog**  **Po/w^c^** | **H-bond donor^d^** | **H-bond acceptor^e^** | **QPlog**  **HERG^f^** | **QPPCaco^g^** | **QPlog**  **khsa^h^** | **rtvFG^i^** | **% human oral absorption^j^** |
| 1 | Madecassoside | 3 | 975.13 | -2.45 | 13 | 32.6 | -4.44 | 1.69 | -1.58 | 3 | 0 |
| 2 | Asiaticoside | 3 | 959.13 | -1.61 | 12 | 30.9 | -5.22 | 2.12 | -1.47 | 3 | 0 |
| 3 | Glycyrrhizin | 3 | 822.94 | 1.75 | 6 | 21.3 | 0.07 | 0.00 | -0.68 | 2 | 0 |
| 4 | Gymnemic acid I | 3 | 806.98 | 3.14 | 6 | 18.6 | -3.73 | 1.26 | 0.13 | 3 | 8.29 |
| 5 | Tangulic acid | 2 | 518.68 | 5.14 | 3 | 6.1 | -0.24 | 7.42 | 0.73 | 0 | 46.73 |
| 6 | 2a,3a,19-trihydroxy-olean-12-en-23,28-dioic acid | 1 | 518.68 | 4.13 | 4 | 8.1 | -0.23 | 5.19 | 0.36 | 0 | 50.98 |
| 7 | 2a,3a,24-trihydroxyurs-12,20(30)-dien-28-oic acid | 0 | 486.69 | 4.02 | 4 | 7.1 | -1.89 | 48.51 | 0.57 | 0 | 80.68 |
| 8 | Bartogenic acid | 1 | 518.68 | 4.22 | 4 | 8.1 | -0.08 | 6.15 | 0.38 | 0 | 52.81 |
| 9 | 19 epibartogenic acid | 1 | 518.68 | 4.24 | 4 | 8.1 | -0.31 | 5.68 | 0.39 | 0 | 52.36 |
| 10 | Corosolic acid | 1 | 472.70 | 5.20 | 3 | 5.4 | -1.73 | 152.24 | 1.01 | 0 | 83.53 |
| 11 | Tanginol | 2 | 506.72 | 2.24 | 6 | 10.2 | -3.61 | 170.25 | 0.16 | 0 | 54.09 |
| 12 | Arjunoic acid | 0 | 488.70 | 4.31 | 4 | 7.1 | -1.75 | 76.10 | 0.64 | 0 | 85.88 |
| 13 | Anhydrobartogenic acid | 2 | 500.67 | 5.04 | 3 | 6.4 | -0.45 | 7.38 | 0.72 | 0 | 46.11 |
| 14 | Tiarellic acid | 1 | 472.70 | 5.28 | 3 | 5.4 | -1.81 | 211.60 | 0.90 | 0 | 86.53 |
| 15 | Boswellic acid 2c | 1 | 498.745 | 7.09 | 1 | 4 | -2.29 | 267.94 | 1.78 | 1 | 100 |
| 16 | Madeccassic acid | 1 | 504.70 | 3.22 | 5 | 8.8 | -1.91 | 27.41 | 0.30 | 0 | 58.61 |
| 17 | Boswellic acid 2a | 1 | 456.70 | 6.73 | 1 | 2.7 | -1.82 | 244.05 | 1.67 | 0 | 96.14 |
| 18 | Katonic acid | 1 | 456.70 | 6.08 | 2 | 3.7 | -1.97 | 169.43 | 1.42 | 0 | 89.53 |
| 19 | Bayogenin | 0 | 488.70 | 4.17 | 4 | 7.1 | -2.06 | 48.12 | 0.63 | 0 | 81.49 |
| 20 | Boswellic acid 2e | 2 | 512.728 | 6.04 | 1 | 6 | -2.32 | 124.45 | 1.39 | 1 | 73.91 |
| 21 | Emarginellic acid | 1 | 488.70 | 5.01 | 3 | 5.1 | -1.92 | 43.73 | 1.00 | 0 | 72.73 |
| 22 | Asiatic acid | 0 | 488.70 | 4.08 | 4 | 7.1 | -1.93 | 47.15 | 0.61 | 0 | 80.80 |
| 23 | Hederagenin | 1 | 472.70 | 5.22 | 3 | 5.4 | -1.86 | 131.07 | 0.99 | 0 | 82.48 |
| 24 | Hyptatic acid A | 0 | 488.70 | 4.50 | 4 | 7.1 | -2.19 | 77.66 | 0.53 | 0 | 87.13 |
| 25 | 3b-trans-sinapoyloxylup-20(29)-en-28-ol | 2 | 634.89 | 8.32 | 2 | 5.9 | -5.76 | 794.30 | 2.30 | 1 | 100 |
| 26 | Phytolaccagenin | 1 | 532.71 | 3.48 | 4 | 9.1 | -2.14 | 12.11 | 0.49 | 0 | 53.80 |
| 27 | Rehmannic acid | 2 | 552.79 | 6.80 | 1 | 6 | -2.23 | 154.16 | 1.64 | 1 | 80.03 |
| 28 | Polygalacic acid | 1 | 504.70 | 3.84 | 4 | 7.8 | -1.78 | 29.60 | 0.53 | 0 | 62.84 |
| 29 | Bryonolic acid | 1 | 456.70 | 5.88 | 2 | 3.7 | -1.41 | 170.65 | 1.35 | 0 | 88.41 |
| 30 | 3b-trans-feruloyloxy-16b-hydroxylup-20(29)-ene | 2 | 604.86 | 8.15 | 2 | 5.2 | -5.78 | 672.89 | 2.34 | 1 | 100 |
| 31 | Morolic acid | 1 | 456.70 | 6.84 | 1 | 2.7 | -1.89 | 250.92 | 1.72 | 0 | 96.99 |
| 32 | Acutangulic acid | 1 | 488.70 | 5.25 | 3 | 5.1 | -1.90 | 108.52 | 1.02 | 0 | 81.16 |
| 33 | Glycyrrhetic acid | 1 | 470.69 | 5.14 | 2 | 5.7 | -1.80 | 118.85 | 1.05 | 0 | 81.24 |
| 34 | 3-O-acetylbetulinic acid | 1 | 498.74 | 7.10 | 1 | 4 | -2.42 | 242.11 | 1.76 | 1 | 100 |
| 35 | Alphitolic acid | 1 | 470.69 | 5.13 | 3 | 5.4 | -2.04 | 120.36 | 0.95 | 0 | 81.26 |
| 36 | Uvaol | 1 | 442.72 | 5.95 | 2 | 3.4 | -3.67 | 2064.78 | 1.56 | 0 | 100 |
| 37 | Coussaric acid | 0 | 486.69 | 4.59 | 4 | 6.1 | -1.83 | 103.76 | 0.70 | 0 | 89.94 |
| 38 | Euscaphic acid (Tormantic acid) | 0 | 488.70 | 4.69 | 4 | 6.1 | -2.47 | 58.92 | 0.85 | 0 | 86.10 |
| 39 | Barrinic acid | 1 | 518.68 | 4.03 | 4 | 8.1 | -0.08 | 4.558 | 0.34 | 0 | 49.38 |
| 40 | 23-epoxy-friedelan-28-oic acid | 1 | 456.70 | 6.21 | 1 | 4 | -1.44 | 599.26 | 1.35 | 0 | 100 |
| 41 | Boswellic acid 2d | 1 | 498.745 | 7.15 | 1 | 4 | -2.40 | 266.60 | 1.81 | 1 | 100 |
| 42 | Barrigenic acid | 1 | 518.68 | 4.03 | 4 | 8.1 | -0.09 | 4.652 | 0.34 | 0 | 49.55 |
| 43 | Imberbic acid | 1 | 472.70 | 5.15 | 3 | 5.4 | -1.90 | 106.21 | 0.98 | 0 | 84.41 |
| 44 | Sumaresinolic acid | 1 | 472.70 | 5.29 | 3 | 5.4 | -2.05 | 171.93 | 1.02 | 0 | 84.97 |
| 45 | Oleanolic acid | 1 | 456.70 | 6.24 | 2 | 3.7 | -1.92 | 264.47 | 1.42 | 0 | 93.89 |
| 46 | Betulinic acid | 1 | 456.70 | 6.19 | 2 | 3.7 | -1.97 | 282.77 | 1.35 | 0 | 94.15 |
| 47 | Rotundic acid | 0 | 488.70 | 4.63 | 4 | 6.1 | -1.63 | 102.09 | 0.74 | 0 | 90.06 |
| 48 | Amooranin | 1 | 470.69 | 5.35 | 2 | 5.7 | -1.66 | 214.37 | 1.02 | 0 | 87.05 |
| 49 | 3 beta acetoxy alfa amyrin | 1 | 454.73 | 7.83 | 0 | 2 | -4.32 | 4278.41 | 2.38 | 1 | 100 |
| 50 | Faradiol | 1 | 442.72 | 5.97 | 2 | 3.4 | -3.79 | 2387.16 | 1.58 | 0 | 100 |
| 51 | Erythrodiol | 1 | 442.72 | 5.95 | 2 | 3.4 | -3.81 | 1810.46 | 1.57 | 0 | 100 |
| 52 | Crotalic acid | 1 | 456.70 | 6.26 | 2 | 3.7 | -1.84 | 312.65 | 1.37 | 0 | 95.30 |
| 53 | Ursolic acid | 1 | 456.70 | 6.15 | 2 | 3.7 | -1.86 | 278.45 | 1.38 | 0 | 93.75 |
| 54 | Germanicol | 1 | 426.72 | 7.09 | 1 | 1.7 | -3.66 | 4429.04 | 2.07 | 0 | 100 |
| 55 | Betulin | 1 | 442.72 | 5.91 | 2 | 3.4 | -3.88 | 1722.40 | 1.51 | 0 | 100 |
| 56 | Pomolic acid | 1 | 472.70 | 5.63 | 3 | 4.4 | -1.58 | 247.94 | 1.13 | 0 | 89.85 |
| 57 | Augustic acid | 1 | 472.70 | 5.29 | 3 | 5.4 | -1.72 | 227.63 | 0.99 | 0 | 87.14 |
| 58 | Lupeol | 1 | 426.72 | 7.31 | 1 | 1.7 | -4.38 | 4428.99 | 2.12 | 0 | 100 |
| 59 | Taraxasterol acetate | 1 | 468.76 | 7.92 | 0 | 2 | -4.26 | 3997.10 | 2.42 | 1 | 100 |
| 60 | Taraxerol acetate | 1 | 468.76 | 7.96 | 0 | 2 | -4.24 | 3976.22 | 2.45 | 1 | 100 |
| 61 | 3beta - acetoxy beta –amyrin | 1 | 454.73 | 7.74 | 0 | 2 | -4.12 | 3949.97 | 2.35 | 1 | 100 |
| 62 | Arnidiol | 1 | 442.724 | 5.95 | 2 | 3.4 | -3.61 | 2030.44 | 1.59 | 0 | 100 |
| 63 | CDDO | 1 | 491.66 | 5.27 | 1 | 7 | -2.31 | 154.36 | 0.99 | 0 | 84.01 |
| 64 | Betunaldehyde | 1 | 440.708 | 5.89 | 1 | 3.7 | -3.63 | 1411.93 | 1.57 | 0 | 100 |
| 65 | Boswellic acid 2b | 1 | 456.70 | 6.80 | 1 | 2.7 | -1.94 | 244.58 | 1.70 | 0 | 96.58 |
| 66 | Maniladiol | 1 | 442.72 | 5.95 | 2 | 3.4 | -3.66 | 2292.18 | 1.58 | 0 | 100 |
| 67 | Acetylursolic acid | 1 | 454.735 | 7.50 | 1 | 2 | -1.83 | 633.58 | 1.93 | 0 | 100 |
| 68 | Boswellic acid 2f | 1 | 426.72 | 7.00 | 1 | 1.7 | -3.53 | 4444.80 | 2.04 | 0 | 100 |
| 69 | Epifriedelanol | 1 | 428.74 | 6.95 | 1 | 1.7 | -3.56 | 3877.42 | 2.03 | 0 | 100 |
| 70 | 20-Epibryonolic acid | 1 | 456.70 | 6.16 | 2 | 3.7 | -1.75 | 224.07 | 1.42 | 0 | 92.15 |
| 71 | Heliantriol C | 0 | 458.72 | 4.99 | 3 | 5.1 | -3.69 | 1316.70 | 1.18 | 0 | 100 |
| 72 | Canophyllal | 1 | 440.70 | 5.74 | 0 | 4 | -3.52 | 1579.54 | 1.49 | 1 | 100 |
| 73 | Beta amyrin | 1 | 426.72 | 7.05 | 1 | 1.7 | -3.73 | 4444.458 | 2.05 | 0 | 100 |
| 74 | Friedeline | 1 | 426.72 | 6.94 | 0 | 2 | -3.48 | 4095.73 | 2.04 | 1 | 100 |
| 75 | Lupeol acetate | 1 | 424.752 | 9.39 | 0 | 0 | -3.87 | 9906.03 | 2.67 | 0 | 100 |
| 76 | Ursonic acid | 1 | 454.69 | 6.29 | 1 | 4 | -1.81 | 373.48 | 1.44 | 0 | 96.90 |
| 77 | Heliantriol B | 0 | 458.72 | 4.94 | 3 | 5.1 | -3.90 | 985.06 | 1.15 | 0 | 100 |
| 78 | CDDO methyl ester | 1 | 505.69 | 4.47 | 0 | 7.5 | -4.22 | 301.38 | 0.93 | 0 | 84.54 |
| 79 | Impressic acid | 1 | 472.68 | 5.27 | 3 | 5.4 | -2.08 | 162.32 | 0.97 | 0 | 82.76 |
| 80 | Boswellic acid 2g | 1 | 440.75 | 7.11 | 1 | 1.7 | -3.64 | 4464.80 | 2.13 | 0 | 100 |

Compounds that follow the softened Lipinski’s rule of five = Green fields; compounds that violate the softened Lipinski’s rule of five = Yellow fields. ^a^Number of violations of Lipinski’s rule of five (Maximum 4), ^b^Molecular weight (< 500 dalton), ^c^Predicted octanol/water partition co-efficient log P (suggested range: -2.0 to 6.5), ^d^Hydrogen bond donors (< 5), ^e^Hydrogen bond acceptors (< 10), ^f^Predicted IC_50_ value for blockage of HERG K+ channels (concern below 5), ^g^Predicted Caco-2 cell permeability in nm/sec (suggested range: < 25 is Poor & > 500 is great), ^h^Predicted binding to human serum albumin (suggested range: -1.5 to 1.5), ^i^Number of reactive functional groups which leads to false positives in HTS assays (0 to 2), ^j^Predicted human oral absorption on 0 to 100% scale (< 25 is Poor & > 80 is great).
